# Mitochondrial genome variation and prostate cancer: a review of the mutational landscape and application to clinical management

**DOI:** 10.18632/oncotarget.19926

**Published:** 2017-08-04

**Authors:** Anton M.F. Kalsbeek, Eva K.F. Chan, Niall M. Corcoran, Christopher M. Hovens, Vanessa M. Hayes

**Affiliations:** ^1^ Laboratory for Human Comparative and Prostate Cancer Genomics, Genomics and Epigenetics Division, Garvan Institute of Medical Research, Darlinghurst, New South Wales, Australia; ^2^ Medical Faculty, University of New South Wales, Randwick, New South Wales, Australia; ^3^ Australian Prostate Cancer Research Centre Epworth, Richmond, Victoria, Australia; ^4^ Departments of Urology and Surgery, Royal Melbourne Hospital, University of Melbourne, Melbourne, Victoria, Australia; ^5^ Central Clinical School, University of Sydney, Camperdown, New South Wales, Australia

**Keywords:** prostate cancer, mitochondrial genome, mtDNA variation, biomarkers

## Abstract

Prostate cancer is a genetic disease. While next generation sequencing has allowed for the emergence of molecular taxonomy, classification is restricted to the nuclear genome. Mutations within the maternally inherited mitochondrial genome are known to impact cancer pathogenesis, as a result of disturbances in energy metabolism and apoptosis. With a higher mutation rate, limited repair and increased copy number compared to the nuclear genome, the clinical relevance of mitochondrial DNA (mtDNA) variation requires deeper exploration. Here we provide a systematic review of the landscape of prostate cancer associated mtDNA variation. While the jury is still out on the association between inherited mtDNA variation and prostate cancer risk, we collate a total of 749 uniquely reported prostate cancer associated somatic mutations. Support exists for number of somatic events, extent of heteroplasmy, and rate of recurrence of mtDNA mutations, increasing with disease aggression. While, the predicted pathogenic impact for recurrent prostate cancer associated mutations appears negligible, evidence exists for carcinogenic mutations impacting the cytochrome c oxidase complex and regulating metastasis through elevated reactive oxygen species production. Due to a lack of lethal cohort analyses, we provide additional unpublished data for metastatic disease. Discussing the advantages of mtDNA as a prostate cancer biomarker, we provide a review of current progress of including elevated mtDNA levels, of a large somatic deletion, acquired tRNAs mutations, heteroplasmy and total number of somatic events (mutational load). We confirm via meta-analysis a significant association between mtDNA mutational load and pathological staging at diagnosis or surgery (*p* < 0.0001).

## INTRODUCTION

Mutations in the mitochondrial genome (mtDNA) have been implicated in both the initiation and progression of cancer, including prostate cancer (PCa). Oncogenic mtDNA mutations are hypothesized to play a role in the metabolic shift, from oxidative phosphorylation (OXPHOS) to aerobic glycolysis, referred to as the Warburg effect [1, 2]. A dysregulation of these processes could cause a general instability of mitochondrial homeostasis, thus impacting other mitochondrial functions such as apoptosis and calcium homeostasis [3]. Multiple reviews have addressed the importance of mitochondrial function in cancers [4–7]. As cancer cells require functional mitochondria, it is assumed that a fine balance exists in the type, frequency and number of mtDNA mutations a cancer cell can tolerate. Additionally, the oncogenic impact of acquired mtDNA mutations will depend on any inherited or ancestral mtDNA variation. Each of these distinct categories will be introduced and discussed in further detail in relation to PCa.

PCa is a genetic disease, which has permitted the discovery of clinically relevant genetic-based biomarkers. Although several genetic tests for improving PCa diagnosis and possible prognosis are commercially available (reviewed in [8]), the clinical benefits of these tests are limited. Largely focused on the nuclear genome, these tests include assessments of gene fusion status, *PTEN* loss, gene expression signatures or methylation signatures. In contrast, the maternally inherited mtDNA has largely been overlooked in genome-based PCa biomarker discovery efforts. While there is clear evidence for mitochondrial genomic changes in prostate and other cancers (reviewed in [5, 9]), the extent and impact of this variation to inform clinical management requires thorough investigation. Therefore, we conducted a systematic literature review and meta-analysis to investigate not only the mutational landscape of the mitochondrial genome in PCa, but also the relationship between mtDNA variation and PCa risk, diagnosis and outcomes. We will also discuss potential therapeutic implications and future focus areas within this emerging field.

## PROSTATE CANCER IS A GENETIC DISEASE

There are as yet no significantly verified modifiable risk factors for the prevention or development of PCa [10]. Genetics is the major contributor with a reported 57% (95% CI of 51%–63%) heritability [11]. Specifically, PCa risk is 2.35 times higher than average with an affected father, and even higher for affected brothers, with a diagnosis rate of 3.14 times the population average [12]. There is also significant risk associated with African-American ancestry, with 1.70 and 2.5-fold greater incidence and mortality rates than Americans of European ancestry, respectively [13]. Genome Wide Association Studies (GWAS) have identified a total of 100 PCa susceptibility alleles, with modest effect sizes (odds ratio <1.5), estimated to explain roughly 33% of familial risk in men of European descent [14–16]. These common variants (minor allele frequency ≥ 5%) are largely located within gene-free regions. Fine-mapping has led to the identification of novel independent signals within roughly a quarter of the PCa susceptibility loci [17]. Rare deleterious mutations in highly penetrant genes, such as *BRCA2* (breast cancer associated), and *HOXB13* (homeobox B13)*,* explain only a small fraction (less than 6%) of the missing heritability [18].

Built on the backbone of inherited variability, tumor progression is driven by acquired genomic variation. Next generation sequencing has greatly advanced the ability to sub-classify PCa based on acquired genomic signatures. The most recent analysis of 333 primary prostate tumor exomes by The Cancer Genome Atlas (TCGA) consortium identified seven molecular PCa subtypes based on recurrent oncogenic drivers. Over half involve an *ETS* (erythroblast transformation-specific transcription factor), specifically *ERG, ETV1, ETV4* or *FL11,* gene fusion event, with *TMPRSS2* (coding for a transmembrane serine protease) as the most common fusion partner. Roughly half of the *ETS* fusion-negative tumors present with mutations in *SPOP* (coding for Speckled-type POZ protein)*, FOXA1* (coding for Forkhead box A1) or *IDH1* (coding for isocitrate dehydrogenase-1), leaving 26% PCa unclassified [19]. Additional known recurrent genomic alterations include; (i) mutations within genes coding for the tumor suppressor tumor protein P53 (*TP53*)*,* the mediator complex subunit 12 (*MED12*) and a cell cycle inhibitor (*CDKN1B*), (ii) deletions and loss of function mutations within *PTEN* (phosphatase and tensin homolog), (iii) overexpression of serine peptidase inhibitor, Kazal type 1 (*SPINK1)*, (iv) somatic copy number alterations (SCNAs) frequently involving gains of chromosome 7 and 8q (including the *MYC* oncogene locus) and losses of chromosome 8p (including oncogenes *FGFR1* and *WHSC1L1*), 13q, 16q and 18, and (v) epigenetic changes, such as silencing of genes encoding Src homology 2 domain containing F (*SHF)*, fatty acid hydroxylase domain containing 2 *(FAXDC2)*, glutathione S-transferase pi 1 (*GSTP1)*, zinc finger protein 154 (*ZNF154)*, and Kruppel like factor 8 (*KLF8)*, the latter reviewed in detail elsewhere [20]. Additionally, unlike other epithelial cancers, PCa shows an abundance of large genomic rearrangements in relation to small somatic variants, suggesting a decreased impact of persistent mutagenic exposure [21, 22].

While both inherited and acquired genomic variation has been significantly linked to PCa, previous studies have focused primarily on the nuclear genome, largely ignoring the maternally inherited mitochondrial genome.

## THE MITOCHONDRIAL GENOME

**Figure 1:**
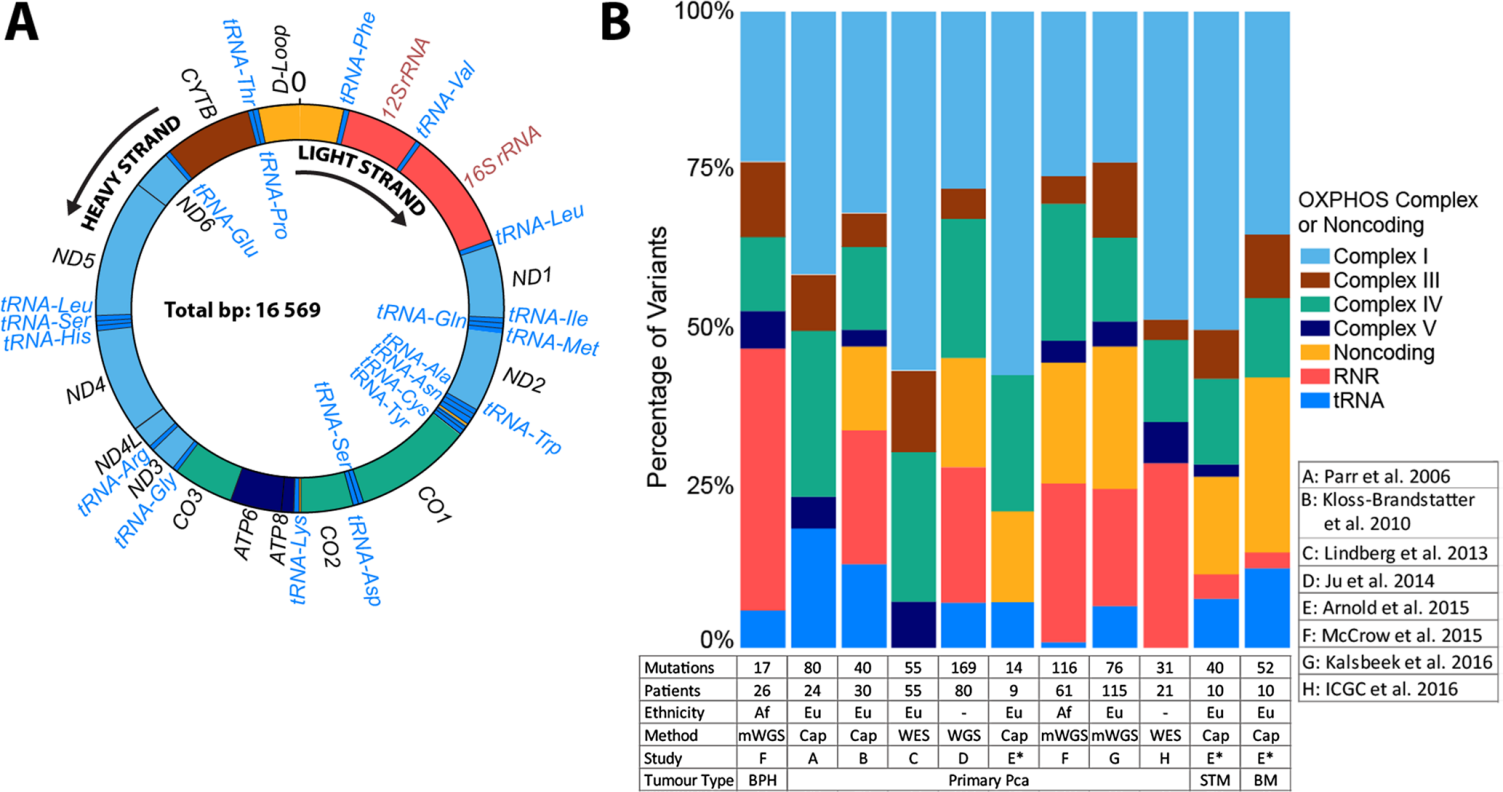
Mitochondrial genome schematic representation and distribution of prostate tissue somatic mutations colored by gene complexes (**A**) Double stranded circular mitochondrial genome depicting the distribution and Heavy (H, outside) or Light (L, inside) strand direction of transcription for 37 genes, 22 tRNAs and 2 rRNAs. (**B**) Distribution of somatic mutations among the OXPHOS complexes and non-coding regions of the mitochondrial genome with numbers below each bar indicating the number of mutations and patients defined by ethnicity, specifically, European (Eu), or African (AF), where whole mitochondrial genome, either by whole genome sequencing (WGS), whole mitochondrial genome sequencing (mWGS) or via amplicon derived capillary sequencing (cap), or coding region analyses, include whole exome sequencing (WES) was performed, which includes a total of eight studies to date. Tissue types include predominantly primary PCa, with smaller studies focused on benign prostate hyperplasia (BPH) or metastatic PCa, specifically bone metastases (BM) or soft tissue metastasis (STM). A single study included patient matched primary and metastatic tissue (*).

Mitochondria are organelles responsible for major cellular processes. These include: ATP production through oxidative phosphorylation (OXPHOS), generating reactive oxygen species (ROS), maintaining calcium homeostasis and controlling apoptosis. Each cell contains hundreds to thousands of mitochondria, each housing tens to hundreds of copies of the circular genome. The 16,569 bases of mtDNA encode 37 genes, including 13 essential proteins that are components of four complexes (I, III, IV, V) of the electron transport chain for OXPHOS and the RNA machinery for assembly of these proteins, two ribosomal RNAs (12S and 16S rRNAs) and 22 tRNAs (Figure 1A). The remaining 7% (1,121 bases) of mtDNA makes up the non-coding control region, also known as the displacement (D)-Loop, where transcription and replication of the mtDNA is initiated. The functional relevance of mtDNA variation can therefore not be ignored.

With efficient DNA-repair mechanisms and lack of protective histones, the mtDNA is prone to mutations, with an approximately 10-fold higher mutation rate than the nuclear genome [23]. The high mutation rate and multiple copies of mtDNA within a cell can lead to a state of heteroplasmy, where not all mitochondrial genomes in a cell or mitochondrion are the same. In contrast to homoplasmy, where all mtDNA molecules are identical, the co-existence of multiple clonal populations of different mitochondrial genomes may contribute significantly to associated clinical heterogeneity. For inherited mitochondrial diseases, it is speculated that the ratio of mutated to wild-type mtDNA required for tissues to reach disease status is 7:3, which is further dependent on both the type of mutation and gene impacted [24–26]. We’ll discuss known mitochondrial mutations and their association with PCa in the following sections.

## INHERITED MITOCHONDRIAL GENOME VARIATION AND PCA

The Prostate Cancer Database Sweden (PCBaSe) has provided substantial evidence that the probability of being diagnosed with PCa is 14.9% in men with one affected brother compared with only 4.8% in the general population [27]. This risk in men with an affected brother over an affected father [12] may suggest a link to the maternally inherited mtDNA. Lack of recombination and strict maternal inheritance of mtDNA, has resulted in the accumulation of inherited mutations defining population-specific mtDNA profiles, known as haplogroups. It has been shown that the variants found in different haplogroups confer different metabolic profiles to cells, suggesting potential haplogroup specific cancer predisposition [28]. Based on demonstrated correlation between population sub-group and PCa risk, with African-Americans having the highest, Europeans intermediate and Asians the lowest risks [29, 30], it is therefore reasonable to postulate that mtDNA haplogroups may provide a susceptibility marker for PCa.

An initial 2006 North American study (*n* = 221 cases, 246 controls), suggested an increased PCa risk with a 1.95 odds ratio for the European-derived haplogroup U, present in 23% of controls and 37% of the PCa patients [31]. This was followed shortly thereafter by a U-haplogroup targeted study (*n* = 71 cases, 128 controls) supporting risk association [32], although later studies were unable to confirm this association (Table 1). While many of these studies were underpowered, a lack of association was also reported from larger studies, including a focused study of 24 European haplogroup defining mtDNA markers in roughly 1000 cases and 500 controls [33] and a mtDNA-wide European study of 620 cases and 616 controls [34]. Similarly, a recent multi-ethnic American study (*n* = 4,086 cases 3,698 controls), including an almost even distribution between African-, Asian-, European- and Latino-Americans, reported no association with haplogroup U in Americans of European ancestry. In the same study, although a nominally significant difference in PCa risk for the Eurasian-derived haplogroup N was found, the investigators noted the false positive rate was greater than 20% after multiple testing correction [35].

**Table 1:** Studies addressing mitochondrial haplogroup associated with prostate cancer risk

		Case	Control		U Haplogroup		N Haplogroup		Largest Representative Haplogroup		
Reference	Ethnic group: Population	No.	No.	Excl. PCa	OR (95% CI)	Power^1^	OR (95% CI)	Power^1^	ID	OR (95% CI)	Power^1^
Booker et al. 2006 [31]^2^	**European**: American	221	246	No	1.95 (1.13–3.38)	83%	-	-	H	0.88 (0.61–1.28)	8%
Kim et al. 2008 [99]	**Asian**: Korean	139	122	Yes	-	NA	0.95 (0.58–1.57)	5%	D	0.96 (0.58–1.6)	5%
Mueller et al. 2009 [100]	**European**: Austrian	304	278	Yes	0.94 (0.61–1.45)	6%	-	-	H	1.14 (0.82–1.59)	10%
Alvarez-Cubero et al. 2012 [95]	**European**: Spanish	239	150	No	1.13 (0.64–1.99)	8%	-	-	H	1.13 (0.75–1.71)	7%
Cano et al. 2014 [101]	**Multi-ethnic**: Colombian	168	140	No	1.11 (0.25–5.02)	5%	0	-	A	0.96 (0.61–1.51)	5%
Fachal et al. 2014 [34]	**European:** Spanish	620	616	no	0.98 (0.76–1.28)	5%	-	-	HV	1.07 (0.85–1.34)	7%
Giorgi et al. 2016 [35]	**Multi-ethnic**: American	4086	3698	Yes	1.02 (0.95–1.12)*	3%	0.90 (0.83–0.98)*	19%	L	1*	-
	European	1004	1003	-	1.07 (0.89–1.28)*	1%		-	L	1*	-
	Asian	1009	999	-	-	-	0.91 (0.85–0.99)*	7%	D	1*	-
	African-American	836	825	-	0.97 (0.91–1.04)*	13%	0.85 (0.74–0.98)*	3%	H	-*	-
	Latino	1099	739	-	1.09 (0.95–1.25)*	2%	-	-	A	1*	-
	Native Hawaiian	138	132	-	-	-	-	-	B	1*	-

Abbreviations: PCa, prostate cancer; OR, Odds Ratio; CI, confidence interval.

^1^Power calculations performed using a genetic power calculator [102] assuming a population PCa prevalence of 0.000548 [103], using the haplogroup frequencies as reported in the studies.

^2^U-haplogroup targeted analysis for 71 cases and 128 controls (excluded for PCa) reported an association with PCa (OR = 2.75, 95% CI 1.2–6.3) [32].

*Values as reported by study.

The question that remains is whether increased PCa risk observed in men with African heritage, may be associated with the presentation of earlier derived mitochondrial haplogroups common within Africa. The earliest diverged haplogroups within Africa display the greatest within population and between sub-population mitochondrial genome variation [36]. One study, although limited in numbers (*n* = 87, including 26 non-cancer prostate samples) found that the earliest diverged mitochondrial haplogroup, L0, specifically L0d from southern Africa, presented with higher pathology grade PCa than those with more recently diverging (non-L0) haplogroups (mean Gleason Score 6.3 versus mean Gleason Score 4.9, *p =* 0.049) [37]. Given this tantalizing result and the known ethnic disparity in PCa, more in-depth investigation seems warranted.

In addition to haplogroup-defining mutations, other population-based inherited mutations have also been implicated in PCa risk. In particular, a 2005 study suggested that *CO1* missense mutations, common within the general European population, increased a man’s risk for PCa [38]. The most common include non-synonymous polymorphisms, 6253 T > C (M117T), 6261 G > A (A120T), 6340 C > T (T146I) and 6663 A > G (I254V). A second study led by the same team, found no evidence that *CO1* mutations play a role in PCa risk in African American men [37], while the previously mentioned multi-ethnic study conversely found the 6253 T > C polymorphism to be nominally protective in 1,661 European and 2,007 African Americans [35]. To truly evaluate an association between mtDNA haplogroups and/or *CO1* polymorphic mtDNA variation and PCa risk and/or outcomes requires significantly larger studies.

## TOTAL SOMATIC MITOCHONDRIAL GENOME BURDEN IN PCA

Compared with the nuclear genome, the mitochondrial genome shows a 55 times higher mutation rate in PCa [39]. Utilizing the TCGA data resource of 1,675 tumors from 31 tumor types, considerable difference in the number of acquired mtDNA mutations per patient (ranging from 0 to 7, 31.1% > 2 variants) and position of mutations was reported [40]. PCa had the largest total somatic mtDNA mutational burden after gastric and hepatocellular carcinoma, with a predominance of single nucleotide variants (SNVs) (*n* = 154) over smaller insertion and deletions (INDELs) (*n* = 15) identified in 80 prostate tumors. Interestingly, this study showed that somatic mtDNA mutations are more likely a direct result of mtDNA replication error, contrary to the expectation that mtDNA mutations predominately result from external mutagens such as ROS. Additionally, missense mutations appeared to be largely heteroplasmic, suggesting negative selection of functionally deleterious mtDNA mutations.

A review of the literature as at 23 December 2016 using the search terms “mitochondrial genome” and “prostate cancer” led to the identification of eight published studies that met the inclusion criteria for whole genome or coding region mtDNA sequencing in PCa, including data deposited into the International Cancer Genome Consortium (ICGC) database (Figure 1B). Five of the studies performed tissue-normal matched whole mtDNA sequencing (mWGS) via targeted next generation sequencing or multi-fragment capillary sequencing (cap) in men from USA (*n* = 10) [41], Canada (*n* = 24) [42], Austria (*n* = 30) [43], South Africa (*n* = 87) [37] and Australia (*n* = 115) [44]. Three analyzed the mtDNA generated by whole exome sequencing (WES) (93% coding region) or whole genome sequencing (WGS) in PCa patients from Austria (*n* = 55) [39] or as part of the international TCGA (*n* = 80) [40] and ICGC (*n* = 21) [45] sequencing efforts, respectively. While most studies focused on primary PCa tissue, the South African study included prostate tissue from men with benign prostate hyperplasia (BPH) (*n* = 26) [37] and the USA study included matched primary (*n* = 9) and both soft (*n* = 10) and bone metastatic tissue (*n* = 10) from the same patients. The eight studies collectively report a total of 635 acquired SNVs and 25 INDELs in 284 of 396 men (71.7%), impacting a total of 680 nucleotide positions (4.2% of the mitochondrial genome).

The total patient mutational burden ranged between 0 and 7 with an average of 1.60 variants per patient for localized disease, which is comparable with other tumor types [40]. The single study to include non-PCa (largely BPH) patient prostate samples, reports significantly fewer somatic variants, specifically 17 in 20 non-PCa patients (0.85 per patient). As the only study from an African ancestral population, this study also reported a higher than average mutational burden for primary PCa, averaging 1.85 somatic mutations per patient [37]. The increased mutational burden could also be as a direct result of more aggressive disease presentation, with almost 70% of the participants presenting with a Gleason score of 8 or greater. The Arnold et al., 2015 study, is the only published report to address somatic mtDNA mutational burden in metastatic PCa [41]. Sequencing the genomes of 10 matched bone and soft tissue metastases, further matched to nine primary prostate tissues, they reported an average of 4.0 and 5.2 somatic mutations, per patient, in soft tissue and bone metastases, respectively. This increase in overall somatic mtDNA mutations relative to those observed in primary PCa, suggests an increase in frequency of somatic mtDNA mutations with tumor progression.

To further assess the total genomic landscape and persistence of mtDNA in PCa metastasis, we analyzed unpublished mitochondrial genome data generated from a published WGS data of seven metastatic PCa patients [46]. This study included nine bone metastases (two patients with multiple metastases) and one neuroendocrine PCa metastasis, as well as three matched primaries and a matched recurrence. The somatic mtDNA mutational burden, with associated frequencies, is summarized in Table 2. Although a low level of mutational heteroplasmy was observed in two of the three primary tumors (two somatic events per patient), none appear to persist within the matched bone metastasis. While overall the acquired mtDNA mutational burden increases with metastasis in these patients, only three mutations approach homoplasmic levels. Further studies are required to determine the overall impact of mtDNA mutations on metastatic PCa.

**Table 2:** Somatic mtDNA mutations observed within metastatic tissue from seven men with prostate cancer derived from WGS data [46]

Patient	Metastasis	Position	Gene	Ref	Alt	Amino Acid change	Impact^1^	Alternate Allele Frequency^2^					
								Primary	Recur	NEC	Bone1	Bone2	Bone3
SM002^3^	Neuroendocrine	1301	RNR1	G	A	NC	NC			0.32			
		6736	COX1	T	C	M278T	NS-Benign			0.49			
		13019	ND5	G	A	G228D	NS-PD			0.97			
SM001	Bone1: pubic Bone2: pelvis	10170	ND3	G	C	E38Q	NS-PD				0.86	0.80	
		16148	D-Loop	C	T	NC	NC				0.82	0.90	
		2440	RNR2	G	A	NC	NC				0.19	absent	
		3492	ND1	A	C	K62N	NS-Benign				0.41	absent	
		8270	Noncoding	C	ins CACCCCCTCT	NC	NC				absent	0.15	
SM067	Bone1: spinal	13513	ND5	G	A	D393N	NS-PD				0.40		
SM068	Bone1: vertebral	316	D-Loop	G	C	NC	NC				0.13		
SM177	Bone1: ilium	567	D-loop	A	AC	NC	NC	0.28			absent		
		6620	COX1	T	C	G239G	Syn	0.26			absent		
		8656	ATP6	A	T	T44S	Benign	absent			0.61^4^		
SM299^5^	Bone1: shoulder	none	-	-	-	-	-	none			none		
SM498	Bone1: sacral Bone2: ilium^6^ Bone3: ilium^6^	3098	RNR2	T	C	NC	NC	0.17	absent		-	absent	-
		14849	CYTB	T	C	S35P	NS-PD	0.10	absent		absent	absent	absent
		13198	ND5	G	A	A288T	Benign	absent	0.38		0.3	0.22	0.31

Abbreviations: Ref, the revised Cambridge Reference Sequence (rCRS) allele; Alt, alternative (mutated) allele; Recur, local recurrence; NEC, neuroendocrine; NC, noncoding; NS, non-synonymous SNV; PD, probably damaging; Syn, Synonymous SNV.

Note: mtDNA sequence data was isolated from published WGS data from seven patients with 21 samples, including neuroendocrine (*n* = 1) or bone metastatic PCa (*n* = 9), with matched whole blood (*n* = 7) and primary PCa tissue (*n* = 3), with a single recurrence (*n* = 1) [46]. Average tumor purity was 61.6% (range 23% to 86%). Total mitochondrial genome coverage ranged from 885 X to 11,403 X.

^1^Impact predicted using PolyPhen-2 [47]. ^2^Frequency of somatic mutations. ^3^A low frequency 0.18 heteroplasmic variant 6787T > C in COI gene was observed in the blood, however, this variant was absent in the matched neuroendocrine tissue from patient SM002. ^4^Mutation also observed in blood from patient SM177 but at lower frequency of 0.31. ^5^No somatic mutations were found in the mtDNA for patient SM299. ^6^Iliac bone metastasis sampled two months apart.

To understand the distribution and therefore the implication of somatic mtDNA mutations in PCa, we classified all reported mutations into mtDNA genic categories. As such we found the regions most impacted are the D-Loop (*n* = 74), the rRNA encoding *RNR2* (*n* = 62), and the *CO1* gene (*n* = 59) (Figure 1B). When corrected for region and gene length, two tRNAs are highly impacted, tRNA-Pro and -Ala, with 103 and 87 mutations per Kb, respectively. This might be exaggerated due to over-correction for the short length of tRNA genes. When grouped together, tRNAs have 26 mutations per Kb. Within the larger regions, the D-Loop remains the most frequently mutated region, followed by *CO3,* RNR2*, CO2* and *CO1* (*n* = 66, 43, 40, 38 and 37, respectively). All three encoded proteins are located in the highly conserved OXPHOS complex IV region. The significance of variants in this complex driving early prostate tumor evolution requires further investigation. Least affected regions after correcting for gene length are *ATP8, ND6,* and *ATP6* (*n* = 5, 23 and 24, respectively) and most other tRNAs, suggesting a potential negative selection for mutations in those genes during tumorigenesis.

One should be mindful that reported differences in number and distribution of somatic variants detected may, at least in part, be a result of different variant calling methods, detection thresholds and/or populations sourced. The lack of rRNA and noncoding variants in the Parr et al. 2006 study, for example, can be attributed to the lower sequencing coverage (78%) and the noted lack of the 12S and 16S rRNA regions due to nuclear mitochondrial DNA segment (numts) co-amplification [42]. For the Lindberg et al., 2013 [39] and ICGC studies [45], data was generated from WGE data and therefore excluded the noncoding region, while the targeted multi-fragment capillary sequencing methods would each present with some level of amplification biases. Overall the WGS and mWGS studies report similar mutational distributions, with the single African study reporting an overall decrease in somatic mutations in the tRNAs compared to European studies.

## PCA ASSOCIATED MITOCHONDRIAL SOMATIC VARIANTS

In addition to the eight whole genome and whole coding region mitochondrial studies for PCa mentioned above, additional studies include partial mtDNA analyses. Taken together, a total of 766 unique somatic mtDNA prostate tissue associated mutations have been identified to date (Supplementary Table 1). Excluding mutations in non-cancerous BPH, the vast majority of the 749 PCa-associated somatic mutations reported are defined as heteroplasmic, with only 14.8% (92/621 with reported heteroplasmy) described as homoplasmic. We note an increased frequency of metastatic PCa derived somatic mtDNA variations described as homoplasmic (46.2%, 24/52) compared with primary PCa (11.2%, 64/569). Although none of the non-synonymous homoplasmic mtDNA variants had a predicted damaging impact, using PolyPhen2 [47], their affect on ROS-production requires further investigation. It should be cautioned that all pathogenicity prediction tools have limitations, for example PolyPhen2 is significantly better at detecting loss-of-function over gain-of-function mutations [48]. In our additional analyses, we identify three somatic mutations, two with predicted oncogenic potential, approaching homoplasmic levels in bone metastatic and brain metastatic neuroendocrine PCa (Table 2). Thus, the original observation that the level and degree of heteroplasmy increases with tumor activity, as based on semi-quantitative capillary sequencing data [43], may warrant further investigation.

Of all the somatic mtDNA mutations reported in PCa, 80 sites (9%) were observed in two or more patients (Table 3). Four of these, specifically 303, 16182, 16183 and 16519, are known polymorphic sites and are therefore unlikely to have significant impact on PCa [49]. Of the remaining 76 sites, the most common include: 10398 A > G, 72 T > C and 1171 9G > A, presenting in seven, six and five patients, respectively. The next fourteen most common mutations were reported in three to four patients. Ten occur within the non-coding region, four are synonymous and the three non-synonymous mutations are predicted by PolyPhen2 to be benign. Further evidence would be required, however, to support lack of pathogenicity. Factoring in the cautionary interpretation of pathogenicity, of the 58 somatic mtDNA mutations reported in each of two patients, 15 are predicted by PolyPhen2 to have pathogenic potential. Of interest, two cause premature termination of transcription of *ND1* (3664 G > A) and *CO1* (6930 G > A) genes, and one a frameshift within *ND5* at nucleotide 12417. Reported in primary PCa tissues, the *ND1* and *ND5* variants appearing to be specific to PCa. The *CO1* 6930 G > A variant, which results in loss of the last 170 amino acids of the C-terminal region of the COX1 protein (G343X), has been shown to disrupt the functional structure of Complex IV, causing multisystem mitochondrial disorder when inherited [50]. The other 12 somatic non-synonymous mutations predicted to have pathogenic potential impact the following genes: *ND4* (3), *CO1* (2), *CYTB* (2), *ND5* (2), *ND1* (1), *ND2* (1) and *ND4L* (1), with all reported in primary PCa and one *ND4* 11351 G > A also within a single soft tissue metastasis.

**Table 3:** Recurrent somatic mtDNA mutations reported for prostate cancer (*n* = 80 nucleotide positions)

Position	Gene	Allele	Amino Acid	Prediction	Affected Patients	Tissue	References
10398	ND3	A>G	T114A	NS-Benign	7	BM	[41]
72	D-Loop	T>C	NC	NC	6	PPCa	[104], [40], [37]
11719	ND4	G>A	G320G	Syn	5	PPCa, BM, STM	[39], [41]
16519*	D-Loop	C>C, T>C	NC	NC	5	PPCa, BM, STM	[105], [40], [41], [44]
73	D-Loop	A>G	NC	NC	4	PPCa, BM	[105], [41]
195	D-Loop	C>T, T>C	NC	NC	4	PPCa, STM	[105], [44], [41]
9899	COX3	C>T, T>C	H231H	Syn, Syn	4	BM, STM	[41]
10463	tRNA-Arg	C>T, T>C	NC	NC	4	BM, STM	[41]
16093	D-Loop	C>C, C>T	NC	NC	4	PPCa	[105], [44]
16183*	D-Loop	A>AC, A>C, A>G	NC	NC	4	PPCa, BM	[90], [105], [40], [46]
16189	D-Loop	T>C	NC	NC	4	PPCa	[90], [105], [40]
309*	D-Loop	8C>9C, 8C>7C, 7C>8C	NC	NC	4	PPCa	[43]
4216	ND1	C>T, T>C, UN	Y304H, H304Y, UN	NS-Benign, UN	3	PPCa, BM, STM	[42], [41]
11251	ND4	A>G	L164L	NC	3	BM, STM	[41]
12372	ND5	G>A	L12L	Syn	3	PPCa, STM	[90], [39], [41]
14766	CYTB	C>T	T7I	NS-Benign	3	PPCa, BM, STM	[39], [41]
15928	tRNA-Thr	G>A	NC	NC	3	BM, STM	[41]
16294	D-Loop	C>T	NC	NC	3	PPCa, BM, STM	[41], [37]
16380	D-Loop	C>T	NC	NC	3	PPCa	[37]
303*	D-Loop	2C>C, 8C>7C, 8C>9C, 2C>C	NC	NC	3	PPCa, BM	[105], [104], [46]
64	D-Loop	C>A	NC	NC	2	PPCa	[40], [37]
146	D-Loop	T>C	NC	NC	2	PPCa	[90], [40]
150	D-Loop	C>T	NC	NC	2	PPCa, BM	[105], [41]
152	D-Loop	C>T, T>C	NC	NC	2	PPCa, BM	[41]
185	D-Loop	G>A, A>A	NC	NC	2	PPCa	[37], [44]
189	D-Loop	A>G	NC	NC	2	PPCa	[90], [37]
204	D-Loop	T>C	NC	NC	2	PPCa	[90], [40]
207	D-Loop	G>A	NC	NC	2	PPCa	[90], [105]
214	D-Loop	A>G	NC	NC	2	PPCa	[40], [37]
235	D-Loop	G>C, A>G	NC	NC	2	PPCa	[90], [105]
244	D-Loop	AA>A	NC	NC	2	PPCa	[105]
709	RNR1	G>A	NC	rRNA	2	PPCa	[43], [37]
902	RNR1	G>A	NC	rRNA	2	PPCa	[40], [37]
1339	RNR1	G>A	NC	rRNA	2	PPCa	[40], [44]
1464	RNR1	G>A	NC	rRNA	2	PPCa, STM	[41], [44]
1806	RNR2	T>C	NC	rRNA	2	PPCa	[40]
2819	RNR2	G>A	NC	rRNA	2	PPCa	[40]
3357	ND1	G>A	M17M	Syn	2	PPCa	[90], [37]
3394	ND1	T>C, UN	Y30H, UN	NS-Benign, UN	2	PPCa	[42], [43]
3454	ND1	G>A	A50T	NS-PD	2	PPCa	[40], [37]
3664	ND1	G>A	G120X	Stopgain	2	PPCa	[40]
3915	ND1	G>A	G203G	Syn	2	PPCa	[39], [44]
4522	ND2	T>C	L18P	NS-PD	2	PPCa	[43], [44]
4561	ND2	C>T, T>C	V31A, A31V	NS-Benign	2	PPCa, STM	[40], [41]
4917	ND2	A>G, UN	N150D, UN	NS-Benign, UN	2	PPCa	[42], [39]
5043	ND2	G>A	A192T	NS-Benign	2	PPCa	[39]
5590	tRNA-Ala	G>A	NC	tRNA	2	PPCa	[40]
5894	NC5	A>+C, A>-C	NC	NC	2	PPCa	[40]
6384	COX1	G>A	A161T	NS-PD	2	PPCa	[43], [37]
6930	COX1	G>A	G343X	Stopgain	2	PPCa	[40], [44]
7028	COX1	C>T	A375A	Syn	2	PPCa, BM, STM	[41]
7293	COX1	G>A	A464T	NS-PD	2	PPCa	[40], [37]
7854	COX2	T>C	V90A	NS-Benign	2	PPCa	[40],[45]
8269	COX2	G>G, G>A	X228X	Syn	2	PPCa	[43], [44]
8697	ATP6	G>A, A>G	M57M	Syn	2	PPCa, BM	[39], [41]
9477	COX3	G>C, UN	V91L, UN	NS-Benign, UN	2	PPCa, STM	[42], [41]
10756	ND4L	T>C	L96P	NS-PD	2	PPCa	[37]
11246	ND4	G>A	A163T	NS-PD	2	PPCa	[39], [40]
11351	ND4	G>A	A198T	NS-PD	2	PPCa, STM	[37], [41]
11775	ND4	G>A	S339N	NS-PD	2	PPCa	[40],[45]
11907	ND4	T>C, UN	V383A, UN	NS-Benign, UN	2	PPCa	[42], [40]
12308	tRNA-Leu2	A>G	NC	NC	2	PPCa, STM	[90], [41]
12417	ND5	C>-A, C>+A	L20P	Frameshift	2	PPCa	[40]
12457	ND5	G>A	A41T	NS-PD	2	PPCa	[37], [44]
13367	ND5	G>A	G344E	NS-PD	2	PPCa	[40],[45]
13368	ND5	G>A, A>G	G344G	Syn	2	PPCa, BM	[39], [41]
14560	ND6	A>A, G>A	V38V	Syn	2	PPCa	[37], [44]
15375	CYTB	G>A	G210E	NS-PD	2	PPCa	[40], [37]
15500	CYTB	G>A	D252N	NS-PD	2	PPCa	[40], [37]
15607	CYTB	A>G, G>A	K287K	Syn	2	BM, STM	[41]
16034	D-Loop	G>A	NC	NC	2	PPCa	[40]
16035	D-Loop	G>A	NC	NC	2	PPCa	[37], [44]
16182*	D-Loop	A>C	NC	NC	2	PPCa	[105]
16224	D-Loop	C>C, C>T	NC	NC	2	PPCa, STM	[41], [44]
16290	D-Loop	T>T, C>T	NC	NC	2	PPCa	[37], [44]
16298	D-Loop	T>C	NC	NC	2	PPCa	[105]
16311	D-Loop	C>T, T>C	NC	NC	2	PPCa, STM	[105], [41]
16320	D-Loop	C>T	NC	NC	2	PPCa, STM	[40], [41]
16327	D-Loop	C>T	NC	NC	2	PPCa	[40], [37]
16390	D-Loop	G>A	NC	NC	2	PPCa	[37]

Abbreviations: Pat, patients; UN, Unknown; NC, noncoding; Syn, synonymous; NS, non-synonymous; PD, probably damaging; PPCa, primary prostate cancer; BM, bone metastasis; STM, soft tissue metastasis.

Notes: ^1^UN (unknown) alleles, as Parr et al. 2006 [42] reported only the mutated nucleotide position, not the allele change, thus amino acid change and prediction could not be generated. ^2^Pathogenicity is represented in bold and defined as a stop gain, frameshift, or is predicted as possibly damaging using PolyPhen2 [47]. *Known highly polymorphic mtDNA nucleotides involving a polyC at position 303-315, A/C changes at positions 16182 and 16183, and a C/T changes at position 16519, although reported, should likely not be considered as somatic.

In contrast to primary PCa, acquired mutational hotspots or recurrence of mtDNA has been suggested for metastatic PCa. Specifically, the 10398 A > G (T114A) within *ND3* was reported in 7 of 10 bone metastatic tumors from a single study [41]. This variant was absent in patient matched soft tissue metastasis (lymph node, liver or lung) and primary PCa, while observed in 3 of 21 thyroid carcinomas [51]. In contrast, a low degree of mtDNA 10398G heteroplasmy, or high degree of 10398A heteroplasmy, has been suggestive as a marker for poor prognosis in patients with non-small cell lung cancer [52, 53] and cervical cancer [54], respectively. The 10398 A > G variant was somatically absent in 395 primary PCa’s (Figure 1B), absent in a targeted study of the mtDNA *ND3* gene in 77 Mexican-Mestizo men with aggressive primary PCa [55], and absent in the single neuroendocrine and the nine bone metastases from six PCa patients reported in this review (Table 2) [46]. While the impact of acquiring the G-allele on PCa metastasis requires further confirmation, 10398 is a common haplogroup defining polymorphic site. Specifically, 10398 G > A variant is one of only five known polymorphic markers identifying one of the two major mitochondrial haplogroups that emerged ‘out-of-Africa’ roughly 65,000 years ago. While the ancestral within Africa L3 haplogroup and the out-of-Africa sister haplogroup M is defined by 10398 G-allele, the globally dispersed haplogroup N is defined by 10398 A-allele, with the K1 and J1 branches derived from a known 10398 A > G back mutation [49]. Less common within Africa, the 10398 A-allele has been significantly associated with increased risk for invasive breast cancer in African-American woman (654 cases, 605 controls), yet this significance was absent in White Americans (879 cases, 760 controls) [56]. Additionally, in an African-American derived breast cancer cell model, the mtDNA 10398 G > A variant was shown to increase ROS production, confer resistance to apoptosis and promoted metastasis [57]. As with the somatic 10398 A > G variant, the acquired *ND4* 11719 G > A variant reported in four of 10 metastatic [41] and a single primary PCa [39], is a known polymorphic back mutation defining the H5a1b and H27 European-predominant haplogroups from the original R0 (previously pre-HV haplogroup) defining 11719 A > G polymorphism. The predominance of haplogroup defining polymorphic sites to acquire somatic mtDNA mutations during metastases needs further exploration.

It is well established that mutagenic mechanisms exhibit different mutational DNA signatures. For mtDNA, it has been speculated that mutations would arise from the proximity to ROS species generated by OXPHOS [55]. ROS-induced mutations are characterized by guanine oxidation resulting in an over representation of G:C > T:A transversion mutations [58]*.* However, mutations identified in mtDNA in PCa predominantly involve C > T (57%) and T > C (35.9%) transitions, with only 1.3% G:C > T:A mutations observed. This is comparable to mtDNA mutations in all 1,675 tumor types sequenced as part of the TCGA effort [40], with speculated ROS-associated mutations accounting only for 4% of variants. Furthermore, Ju et al., 2014, found a strong bias of C > T and A > G mutations on the mtDNA heavy strand and G > Aand T > C bias on the light strand in cancer, which holds true for PCa.

Besides SNVs and indels, large mtDNA deletions have been reportedly acquired during prostate tumorigenesis. Using long-range amplification and Southern blot analysis, a 2001 study observed large somatic mtDNA deletions in all 34 primary PCa specimens [59]. Furthermore, they suggest that the number of large deletions observed increases proportionally with the patient’s age. While men in their forties reportedly present with on average 1.25 additional deletions, this increases to 5.9 additional deletions in men in their seventies, including a common 14.8 Kb deletion observed in all men over 60 years. A single group has also reported the acquisition of a common 3,379 basepair mtDNA deletion in PCa [60]. This variant, which knocks out the terminal end of *ND4L,* the complete *ND4*, most of the *ND5* genes, and three tRNAs, was further reported to predict the presence of malignant tumor tissue proximal to biopsies appearing histologically normal [61]. The significance of these large deletions requires further verification.

## BIOLOGICAL IMPACT OF PCA ASSOCIATED MITOCHONDRIAL MUTATIONS

Compared with the nuclear genome, the mitochondrial genome is largely coding. It is well established that mutations in mtDNA contribute to mitochondrial dysfunction as shown in mitochondrial diseases, cell models and cancer [1, 5, 7]. Mitochondrial dysfunction has been implicated in tumor growth, metastatic potential and therapeutic response rate in several cancers [9]. The inherited mtDNA 6124 T > C mutation in the *CO1* gene has been shown to alter reactive oxygen and nitrogen species and proliferation in a patient with PCa by using patient derived cybrid models (a fusion of enucleated patient fibroblasts to 14B rho cell not containing mitochondrial DNA) [62], implicating an effect of variants in the mtDNA on tumorigenesis. Using the same technique, they introduced this mutation in PC3 PCa cells and identified a resistance to the apoptotic effects of statins [63]. Metastatic potential was shown to be altered in PCa cells in the bone microenvironment in cybrid models by inheriting an mtDNA 8993 T > G mutation in the *ATP6* gene [64]. Cybrids allow researchers to introduce mutated mtDNA into a prostate epithelial or cancer cell line and investigate if the mutations change cellular characteristics [65]. These modified cells can be injected into mice and, as shown for lung cancer cells, tumors can acquire metastatic potential from mtDNA replacement from a highly metastatic cell line [66]. These techniques are becoming more reliable and have been implicated in restoring mutated mtDNA in patients carrying inherited mitochondrial disease, with further advances opening the possibility to restore these mutations in cancer patients [67].

Although ROS-induced mtDNA mutations appear to be rare in PCa, based on mutational signatures, both the inherited 6142 T > C and 8993 T > G PCa associated mtDNA mutations have been shown to be ROS-producing [68]. Notably, elevated levels of ROS are evident in numerous cancer types, including PCa [69]. A recent review addresses the functions, and potential mechanisms, of elevated ROS-production in both promoting and suppressing tumor development [70]. Specifically, increased ROS-production has been associated with uncontrolled cell cycle, clonogenicity, invasion and metastasis in PCa cells [71]. Further functional support comes from a study showing that ROS-producing mtDNA mutations play a role in regulating lung-derived tumor cell metastasis, with potential for therapeutic intervention [66]. We have identified 80 recurrent (presenting in two or more patients) somatic PCa associated mtDNA mutations reported to date, with roughly 19% having a predicted impact on protein function. The impact of mtDNA mutations acquired during prostate tumorigenesis on ROS-production, however, remains unknown. Interestingly, Arnold et al., (2009) suggests that OXPHOS complex IV mutations increase ROS levels (data not shown) [64], while in this review we observe an over representation of somatic mutations within the coding genes of this highly conserved complex. The significance of somatic mtDNA mutations driving ROS-production and in turn prostate tumorigenesis, requires further investigation.

## MITOCHONDRIAL DNA COPY NUMBER ALTERATIONS IN PCA

Mitochondrial copy number in normal cells is regulated by ATP demand and hypoxia [72]. Although there is variability in the number of copies of mtDNA in a cell, the mtDNA copy number tends to be heritable, declining with age [73, 74]. mtDNA copy number has been studied in blood and cancer tissue of patients. A meta-analysis of peripheral blood mtDNA copy number in 36 cancer studies found no significant correlation of mtDNA copy number and cancer risk in general. Subgroup analysis showed a positive correlation for risk of lymphoma and breast cancer (OR = 1.645, 95% CI = 1.117–2.421, *P* = 0.012; OR = 1.721, 95% CI = 1.130–2.622, *P* = 0.011) [75]. A meta-analysis investigating cancer prognosis included both blood (*n* = 6) and tissue (*n* = 14) studies and established that a high mtDNA copy number in blood was associated with reduced disease-free survival (HR = 1.582, 95% CI: 1.026–2.439, *p* = 0.038), and a high mtDNA copy number in tissue in contrast was associated with an increased disease free survival (HR = 0.593, 95% CI: 0.411–0.857, *p* = 0.005) [76]. A study investigating urological cancers, including PCa reported an increase in cell free circulating mtDNA in cancer patients [77]. In PCa specific studies, a single retrospective study, although underpowered (193 cases and 194 controls), reported an association between mtDNA copy number in peripheral blood leucocytes and PCa risk [78]. However, this finding was not replicated in a larger prospective nested study (793 cases and 790 controls) [79]. Inverse associations were also found between mtDNA copy number and aggressive and non-aggressive PCa, respectively, which may be a direct result of study design, with the authors calling for independent replication. A study investigating 1751 non-Hispanic white men found that low mtDNA copy number in blood corresponded to a higher risk disease ORs of 1.33 (95% CI, 0.89–1.98; *p* = 0.17) and 1.53 (95% CI, 1.02–2.30; *p* = 0.04), for second and first tertiles respectively and increased risk of disease progression (HR, 1.56; 95% CI, 0.96–2.54; *p* = 0.07) [80].

There is less information available on tissue-derived mtDNA copy number, with one small study reporting a higher mtDNA content in PCa versus normal prostate cells in seven of nine cases [81]. A reduction in mtDNA content in normal, BPH and PCa tissue was reported for American men of African versus European ancestry and in men aged ≥ 60 years [82]. A recent study analyzed TCGA cancer genome data for mtDNA copy number across 22 cancers compared to normal tissue. It observed a trend for several cancers, particularly bladder, breast and kidney, to be depleted of mtDNA and found an association with somatic alteration in the nuclear genome and respiratory gene expression, suggesting that mitochondrial activity was suppressed in these cancers [83]. In contrast and uniquely to PCa, the same study reported an inverse correlation between mtDNA copy number and mitochondrial related nuclear gene expression. The mechanism through which these changes in mitochondrial copy number occur are unclear.

## MITOCHONDRIAL GENOME VARIANCE IN CLINCIAL PRACTICE

Although PCa is the most commonly diagnosed male cancer and has the second highest cancer mortality rate in men living in the most economically developed countries [84], clinical management is complicated by extreme variability in disease course. This includes symptom-free indolent to lethal disease, and a wide range of treatment options, including active surveillance, radiotherapy, hormone therapy, radical prostatectomy, as well as combination therapies. While PCa treatment has a high curability rate [85], radiotherapy and surgery have notable side effects, including erectile dysfunction, urinary incontinence and pain [86], and hormone treatment is associated with decreased libido, obesity and osteoporosis [87]. In addition to digital rectal examination, a suspected diagnosis is reliant on blood-based prostate specific antigen (PSA) testing which, since its introduction in the mid 1990’s, has greatly increased the incidence of PCa with minimal impact on morbidity and mortality [88]. Actual PCa diagnosis can only be made with a prostate biopsy and associated pathological Gleason grading [89]. New non-invasive biomarkers are therefore urgently required to improve clinical management of PCa.

The first acquired mtDNA variant to be implicated in PCa and developed as a commercial biomarker for suggestive false negative biopsy identification is based on the previously described large 3.4 Kb deletion [60]. While this biomarker was implicated in men of European ancestry, it was suggested to be absent in an Australian [44] and an African study [37]. Other suggested mtDNA-based biomarkers requiring further clarification include an elevated level of acquired tRNAs mutations and an increased level of heteroplasmy [39, 43]. Correcting for regional length, we found tRNAs Pro and Ala to be most frequently mutated when considering all whole mitochondrial genome PCa studies. While a trend towards tRNA somatic mutations has been reported in European studies, the single African study reports fewer somatic tRNA mutations than expected [37]. The observation of increased frequency of homoplasmic variation in metastatic over primary PCa when considering all PCa associated mutations and therefore potential as oncogenic drivers, calls for further studies focused on metastatic disease.

**Figure 2:**
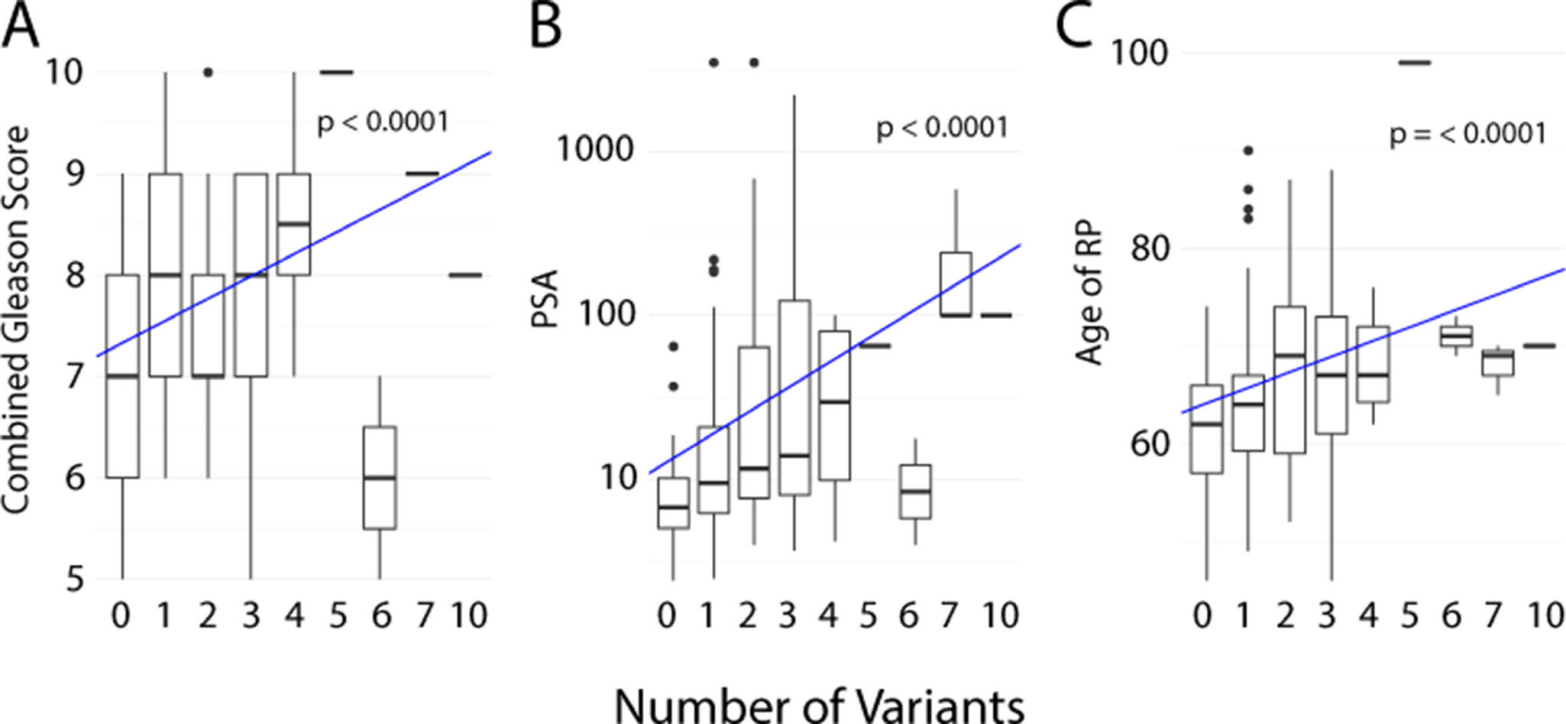
Number of somatic mtDNA variants correlated with clinical characteristics from four studies (**A**) Sample combined Gleason score. (**B**) PSA level before radical prostatectomy or at diagnosis. (**C**) Age of patient at radical prostatectomy or diagnosis. The line and *p*-value represent the linear model for given characteristic by mutation number, generated using R with ggplot2 package [97, 98].

More recently, a correlation between total number of somatic mtDNA variants (defined as mutational load) and Gleason score at diagnosis has been suggested from an African-based [37] and Australian-based study [44]. Furthermore, the latter reported a correlation between a higher mutational load and risk of biochemical relapse after surgery, with a Hazard Ratio (HR) of 2.17 for ≥ 1 SNVs and a HR of 3.82 for ≥ 2 SNV. Both studies noted a lack of recurrent mutations. These studies suggest that rather than a single mtDNA mutation, the total mtDNA mutational burden is a potential biomarker of aggressive disease. Combining the four studies where data was available to associate number of somatic mtDNA mutations with clinical characteristics [37, 39, 43, 44], we show a significant increase in the number of variants with sample combined Gleason score (*p* = < 0.0001) (Figure 2A), with PSA level before radical prostatectomy or at diagnosis (*p* = 0.30) (Figure 2B) and age of patient at radical prostatectomy or diagnosis (*p* = < 0.0001) (Figure 2C), further supporting the proposal that mtDNA mutation burden may be an indicator of PCa. The McCrow et al., 2016 paper further suggests that these mutations may not be causal but rather an indicator of a higher degree of mutagenesis, while also suggesting an upper limitation of cellular tolerance for mutational load [37]. Although less frequent, somatic mtDNA variants have also been identified in tumor-matched urine and plasma from PCa patients [90]. Two other studies revealed circulating cell free tumor mtDNA in PCa and further showed a correlation with survival or early PSA recurrence [91, 92].

The mitochondrial genome has clear advantages over the nuclear genome as a target for PCa biomarker development, including its significantly smaller genome size, increased biological stability and abundance, as well as providing a logical correlation between observed clinical heterogeneity and the uniquely mitochondrial phenomenon of heteroplasmy. As formalin fixation and paraffin embedding (FFPE) of prostate tissue is routine practice during pathological diagnosis, the process itself increases potential for the introduction of mutations and DNA breaks and in turn sequencing error [93]. These FFPE-derived artifacts are less prominent in mtDNA than nuclear DNA due to the higher stability and abundance. The small size and abundance also facilitates the detection of small quantities of cell free mtDNA or circulating tumor cells in diagnostically appropriate bodily fluids such as blood, urine or semen. Additionally, whole exome or genome sequencing efforts often overlook the mitochondrial genome, even though higher sequencing coverage is inadvertently generated relative to the targeted nuclear genome. Advances in analytical software to address heteroplasmy will greatly advance the utilization of the mtDNA data, which is essentially generated as a byproduct of WGS/WES. Although RNA sequencing allows for variant calling of the expressed mtDNA genes, one should be cautious that this does not cover the whole mtDNA and introduces coverage bias through differential RNA half live and potential sequence bias by post transcriptional modifications of RNA [94].

## CONCLUDING REMARKS

In addition to the nuclear genome, the mitochondrial genome not only carries potential to increase a man’s risk for PCa via germline variation, but the maternally inherited genome may also acquire mutations during tumorigenesis. While no single population-specific non-African haplogroup appears to definitively increase PCa risk, further studies would be beneficial to address relevance and potential aggressive disease association in African populations. Furthermore, the observation that inherited mtDNA mutations provides a direct biological impact on mitochondrial function and PCa tumorigenesis, calls for further investigation of the role of germline mtDNA variation in PCa.

While a maximum of seven acquired mtDNA mutations were reported in primary PCa from non-Africans, up to ten mutations were found within a single African study biased towards aggressive disease pathology at presentation. Studies focused on metastatic PCa are limited. A 2015 study and unpublished mtDNA data presented in this review, suggest mutational burden, oncogenic potential, level of heteroplasmy, and recurrence rate for somatic mtDNA mutations increase with metastasis. Overall there is a paucity of evidence for ROS-induced mutagenesis, while meta-analysis for whole mitochondrial genome data shows preferential acquisition of variants in genes coding for the OXPHOS complex IV (cytochrome *c* oxidase, COX), as well as specific regulators (tRNA-Pro, tRNA-Ala and RNR2) and the control region (D-loop). The pathogenic significance for the abundance of somatic mtDNA mutations in the highly conserved COX coding genes (*CO1, CO2*, and *CO3*), which form the core of the complex IV enzyme in which the redox reaction takes place, calls for further investigation. This is further supported by known pathogenetic mutations within this region, either directly associated with mitochondrial diseases [95], or biological impact via altering levels of ROS production [62]. Apart from SNVs and indels, mtDNA large deletions and copy number variants have also been reported to be associated with PCa, although the significance of these variants requires further investigation.

Although still in early stages of discovery for PCa, the significance of the mitochondrial genome to influence cancer progression and cancer therapy is gaining rapid momentum [7]. In particular, the role of mtDNA mutations to induce metastasis via elevating ROS production, provides exciting opportunities for therapeutic intervention via inhibiting ROS signaling [96]. We argue that the hope for preventing and treating clinically heterogeneous PCa lies in the broad application of individualized genomics, which forms the basis of precision medicine. This review provides evidence that supports the inclusion of the mitochondrial genome in the future of PCa precision medicine. The mitochondrial genome provides not only an explanation for extreme PCa clinical heterogeneity via co-expression of inherited and somatic mtDNA variation in varying ratios (heteroplasmy), but has benefits for diagnostic and/or prognostic biomarker development, as well as improving therapeutic intervention

## SUPPLEMENTARY MATERIALS TABLE





## Abbreviations

mtDNA: mitochondrial DNA
PCa: prostate cancer
OXPHOS: oxidative phosphorylation
GWAS: genome wide association study
TCGA: the cancer genome atlas
SCNAs: somatic copy number alterations
ROS: reactive oxygen species
D-loop: displacement loop
SNV: single nucleotide variant
ICGC: International Cancer Genome Consortium
rCRS: revised Cambridge Reference Sequence
PSA: prostate specific antigen
HR: hazard ratio
FFPE: formalin fixation and paraffin embedding
