# Supplementary material for: Mitochondrial genome variation and prostate cancer: a review of the mutational landscape and application to clinical management
Source: Oncotarget. 2017 Aug 4;8(41):71342–57. doi: 10.18632/oncotarget.19926 (PMC5642640; doi:10.18632/oncotarget.19926)
Supplement: Supplementary file 1 [file oncotarget-08-71342-s001.pdf]

Mitochondrial genome variation and prostate cancer: a review of the mutational landscape and application to clinical management

SUPPLEMENTARY MATERIALS

Supplementary Table 1: All unique PCa or prostate tissue associated mtDNA somatic mutations reported to date. See Supplementary_Table_1
