# Supplementary material for: Mitochondrial genome variation and prostate cancer: a review of the mutational landscape and application to clinical management
Source: Oncotarget. 2017 Aug 4;8(41):71342–57. doi: 10.18632/oncotarget.19926 (PMC5642640; doi:10.18632/oncotarget.19926)
Supplement: Supplementary file 2 [file oncotarget-08-71342-s002.docx]

**Supplementary Table 1: All unique PCa or prostate tissue associated mtDNA somatic mutations reported to date**

| POS | Allele Change | Heteroplasmy | Gene | Amino Acid Change | Prediction | Sample | Patient ID | Technique | Author |
| --- | --- | --- | --- | --- | --- | --- | --- | --- | --- |
| 1 | G>C | No | D-Loop | Noncoding | Noncoding | Primary PCA | CH-16 | Targeted Seq | Chen et al. 2002 |
| 64 | C>A | Yes | D-Loop | Noncoding | Noncoding | Primary PCA | JU-94 | Whole mtDNA | Ju et al. 2014 |
| 64 | C>A | Yes | D-Loop | Noncoding | Noncoding | Primary PCA | M30 | Whole mtDNA | McCrow et al. 2015 |
| 65 | TG>T | Yes | D-Loop | Noncoding | Noncoding | Primary PCA | JU-43 | Whole mtDNA | Ju et al. 2014 |
| 66 | G>T | Yes | D-Loop | Noncoding | Noncoding | Primary PCA | JU-43 | Whole mtDNA | Ju et al. 2014 |
| 72 | T>C | Yes | D-Loop | Noncoding | Noncoding | Primary PCA | GZ-01 | Targeted Seq | Gomez-Zaera et al. 2006 |
| 72 | T>C | Yes | D-Loop | Noncoding | Noncoding | Primary PCA | JU-136 | Whole mtDNA | Ju et al. 2014 |
| 72 | T>C | Yes | D-Loop | Noncoding | Noncoding | Primary PCA | JU-153 | Whole mtDNA | Ju et al. 2014 |
| 72 | T>C | Yes | D-Loop | Noncoding | Noncoding | Primary PCA | JU-184 | Whole mtDNA | Ju et al. 2014 |
| 72 | T>C | Yes | D-Loop | Noncoding | Noncoding | Primary PCA | JU-196 | Whole mtDNA | Ju et al. 2014 |
| 72 | T>C | Yes | D-Loop | Noncoding | Noncoding | Primary PCA | M49 | Whole mtDNA | McCrow et al. 2015 |
| 73 | A>G | Yes | D-Loop | Noncoding | Noncoding | Bone Metastasis | AR-050 | Whole mtDNA | Arnold et al. 2015 |
| 73 | A>G | Yes | D-Loop | Noncoding | Noncoding | Primary PCA | CH-06 | Targeted Seq | Chen et al. 2002 |
| 73 | A>G | No | D-Loop | Noncoding | Noncoding | Primary PCA | CH-04 | Targeted Seq | Chen et al. 2002 |
| 73 | A>G | No | D-Loop | Noncoding | Noncoding | Primary PCA | CH-05 | Targeted Seq | Chen et al. 2002 |
| 93 | A>G | Yes | D-Loop | Noncoding | Noncoding | Primary PCA | JU-146 | Whole mtDNA | Ju et al. 2014 |
| 94 | G>A | No | D-Loop | Noncoding | Noncoding | Primary PCA | CH-04 | Targeted Seq | Chen et al. 2002 |
| 103 | G>A | Yes | RNR1 | Noncoding | Noncoding | BPH | Coloured | Whole mtDNA | McCrow et al. 2015 |
| 103 | G>A | Yes | RNR1 | Noncoding | Noncoding | BPH | Ndebele | Whole mtDNA | McCrow et al. 2015 |
| 106 | G>A | No | D-Loop | Noncoding | Noncoding | Primary PCA | CH-04 | Targeted Seq | Chen et al. 2002 |
| 119 | T>C | Yes | D-Loop | Noncoding | Noncoding | Primary PCA | AK-5358 | Whole mtDNA | Kalsbeek et al. 2016 |
| 143 | G>A | Yes | D-Loop | Noncoding | Noncoding | Primary PCA | M21 | Whole mtDNA | McCrow et al. 2015 |
| 146 | T>C | Yes | D-Loop | Noncoding | Noncoding | Primary PCA | JU-44 | Whole mtDNA | Ju et al. 2014 |
| 146 | T>C | No | D-Loop | Noncoding | Noncoding | Primary PCA | JE-01 | Targeted Seq | Jeronimo et al. 2001 |
| 150 | C>T | Yes | D-Loop | Noncoding | Noncoding | Primary PCA | CH-06 | Targeted Seq | Chen et al. 2002 |
| 150 | C>T | No | D-Loop | Noncoding | Noncoding | Bone Metastasis | AR-380 | Whole mtDNA | Arnold et al. 2015 |
| 152 | C>T | Yes | D-Loop | Noncoding | Noncoding | Bone Metastasis | AR-050 | Whole mtDNA | Arnold et al. 2015 |
| 152 | T>C | No | D-Loop | Noncoding | Noncoding | Bone Metastasis | AR-380 | Whole mtDNA | Arnold et al. 2015 |
| 174 | C>T | Yes | D-Loop | Noncoding | Noncoding | Primary PCA | CH-13 | Targeted Seq | Chen et al. 2002 |
| 185 | G>A | Yes | D-Loop | Noncoding | Noncoding | Primary PCA | AK-13104 | Whole mtDNA | Kalsbeek et al. 2016 |
| 185 | G>A | Yes | D-Loop | Noncoding | Noncoding | Primary PCA | M36 | Whole mtDNA | McCrow et al. 2015 |
| 189 | A>G | Yes | RNR1 | Noncoding | Noncoding | BPH | Swati | Whole mtDNA | McCrow et al. 2015 |
| 189 | A>G | No | D-Loop | Noncoding | Noncoding | Primary PCA | JE-01 | Targeted Seq | Jeronimo et al. 2001 |
| 189 | A>G | No | D-Loop | Noncoding | Noncoding | Primary PCA | M30 | Whole mtDNA | McCrow et al. 2015 |
| 195 | T>C | Yes | D-Loop | Noncoding | Noncoding | Primary PCA | AR-069 | Whole mtDNA | Arnold et al. 2015 |
| 195 | T>C | Yes | D-Loop | Noncoding | Noncoding | Primary PCA | CH-06 | Targeted Seq | Chen et al. 2002 |
| 195 | T>C | Yes | D-Loop | Noncoding | Noncoding | Primary PCA | CH-13 | Targeted Seq | Chen et al. 2002 |
| 195 | T>C | Yes | D-Loop | Noncoding | Noncoding | Soft Tissue Metastasis | AR-069 | Whole mtDNA | Arnold et al. 2015 |
| 195 | C>T | No | D-Loop | Noncoding | Noncoding | Primary PCA | AK-11129 | Whole mtDNA | Kalsbeek et al. 2016 |
| 203 | G>A | Yes | D-Loop | Noncoding | Noncoding | Primary PCA | JU-155 | Whole mtDNA | Ju et al. 2014 |
| 204 | T>C | Yes | D-Loop | Noncoding | Noncoding | Primary PCA | JU-193 | Whole mtDNA | Ju et al. 2014 |
| 204 | T>C | No | D-Loop | Noncoding | Noncoding | Primary PCA | JE-01 | Targeted Seq | Jeronimo et al. 2001 |
| 206 | T>C | Yes | D-Loop | Noncoding | Noncoding | Primary PCA | NA | Targeted Seq | Chen et al. 2002 |
| 207 | G>A | No | D-Loop | Noncoding | Noncoding | Primary PCA | CH-04 | Targeted Seq | Chen et al. 2002 |
| 207 | G>A | No | D-Loop | Noncoding | Noncoding | Primary PCA | JE-01 | Targeted Seq | Jeronimo et al. 2001 |
| 214 | A>G | Yes | D-Loop | Noncoding | Noncoding | Primary PCA | JU-201 | Whole mtDNA | Ju et al. 2014 |
| 214 | A>G | Yes | D-Loop | Noncoding | Noncoding | Primary PCA | M11 | Whole mtDNA | McCrow et al. 2015 |
| 217 | T>C | No | D-Loop | Noncoding | Noncoding | Bone Metastasis | AR-006 | Whole mtDNA | Arnold et al. 2015 |
| 235 | G>C | No | D-Loop | Noncoding | Noncoding | Primary PCA | CH-11 | Targeted Seq | Chen et al. 2002 |
| 235 | A>G | No | D-Loop | Noncoding | Noncoding | Primary PCA | JE-01 | Targeted Seq | Jeronimo et al. 2001 |
| 242 | C>CT | No | D-Loop | Noncoding | Noncoding | Primary PCA | GZ-08 | Targeted Seq | Gomez-Zaera et al. 2006 |
| 244 | AA>A | Yes | D-Loop | Noncoding | Noncoding | Primary PCA | CH-06 | Targeted Seq | Chen et al. 2002 |
| 244 | AA>A | No | D-Loop | Noncoding | Noncoding | Primary PCA | CH-05 | Targeted Seq | Chen et al. 2002 |
| 303 | CC>C | Yes | D-Loop | Noncoding | Noncoding | Bone Metastasis | CH-02 | Whole mtDNA | Hovens et al. 2017 |
| 303 | CC>C | Yes | D-Loop | Noncoding | Noncoding | Bone Metastasis | CH-04 | Whole mtDNA | Hovens et al. 2017 |
| 303 | CC>C | Yes | D-Loop | Noncoding | Noncoding | Bone Metastasis | CH-05 | Whole mtDNA | Hovens et al. 2017 |
| 303 | CC>C | Yes | D-Loop | Noncoding | Noncoding | Bone Metastasis | CH-06 | Whole mtDNA | Hovens et al. 2017 |
| 303 | CC>C | Yes | D-Loop | Noncoding | Noncoding | Bone Metastasis | CH-07 | Whole mtDNA | Hovens et al. 2017 |
| 303 | CC>C | Yes | D-Loop | Noncoding | Noncoding | Bone Metastasis | CH-09 | Whole mtDNA | Hovens et al. 2017 |
| 303 | CC>C | Yes | D-Loop | Noncoding | Noncoding | Bone Metastasis | CH-15 | Whole mtDNA | Hovens et al. 2017 |
| 303 | CC>C | No | D-Loop | Noncoding | Noncoding | Primary PCA | CH-11 | Targeted Seq | Chen et al. 2002 |
| 303 | 8C>7C | No | D-Loop | Noncoding | Noncoding | Primary PCA | GZ-08 | Targeted Seq | Gomez-Zaera et al. 2006 |
| 303 | 8C>9C | No | D-Loop | Noncoding | Noncoding | Primary PCA | GZ-09 | Targeted Seq | Gomez-Zaera et al. 2006 |
| 308 | C>T | Yes | D-Loop | Noncoding | Noncoding | Primary PCA | AK-5349 | Whole mtDNA | Kalsbeek et al. 2016 |
| 309 | 8C>9C | Yes | D-Loop | Noncoding | Noncoding | Primary PCA | KB-PCA017 | Whole mtDNA | Kloss-Brandstatter et al. 2010 |
| 309 | 8C>7C | Yes | D-Loop | Noncoding | Noncoding | Primary PCA | KB-PCA019 | Whole mtDNA | Kloss-Brandstatter et al. 2010 |
| 309 | 8C>7C | Yes | D-Loop | Noncoding | Noncoding | Primary PCA | KB-PCA020 | Whole mtDNA | Kloss-Brandstatter et al. 2010 |
| 309 | 7C>8C | Yes | D-Loop | Noncoding | Noncoding | Primary PCA | KB-PCA027 | Whole mtDNA | Kloss-Brandstatter et al. 2010 |
| 310 | T>C | Yes | D-Loop | Noncoding | Noncoding | Primary PCA | GZ-02 | Targeted Seq | Gomez-Zaera et al. 2006 |
| 313 | C>T | Yes | D-Loop | Noncoding | Noncoding | Primary PCA | CH-10 | Targeted Seq | Chen et al. 2002 |
| 315 | C>T | No | D-Loop | Noncoding | Noncoding | Primary PCA | CH-11 | Targeted Seq | Chen et al. 2002 |
| 316 | G>C | Yes | D-Loop | Noncoding | Noncoding | Bone Metastasis | SM068 | Whole mtDNA | Hovens et al. 2017 |
| 316 | G>C | Yes | D-Loop | Noncoding | Noncoding | Primary PCA | AK-11538 | Whole mtDNA | Kalsbeek et al. 2016 |
| 319 | T>C | Yes | D-Loop | Noncoding | Noncoding | Primary PCA | AK-11538 | Whole mtDNA | Kalsbeek et al. 2016 |
| 408 | T>A | Yes | D-Loop | Noncoding | Noncoding | Primary PCA | M30 | Whole mtDNA | McCrow et al. 2015 |
| 437 | C>T | NA | D-Loop | Noncoding | Noncoding | Primary PCA | NA | Targeted Seq | Chen et al. 2002 |
| 456 | C>T | Yes | D-Loop | Noncoding | Noncoding | Primary PCA | JU-176 | Whole mtDNA | Ju et al. 2014 |
| 459 | C>Del | Yes | D-Loop | Noncoding | Noncoding | Primary PCA | JU-176 | Whole mtDNA | Ju et al. 2014 |
| 487 | A>G | NA | D-Loop | Noncoding | Noncoding | Primary PCA | NA | Targeted Seq | Chen et al. 2002 |
| 489 | T>C | No | D-Loop | Noncoding | Noncoding | Primary PCA | CH-05 | Targeted Seq | Chen et al. 2002 |
| 499 | G>A | No | D-Loop | Noncoding | Noncoding | Primary PCA | CH-04 | Targeted Seq | Chen et al. 2002 |
| 507 | T>C | Yes | D-Loop | Noncoding | Noncoding | Primary PCA | JU-134 | Whole mtDNA | Ju et al. 2014 |
| 513 | G>GCA | Yes | D-Loop | Noncoding | Noncoding | Primary PCA | *JU-46* | Whole mtDNA | Ju et al. 2014 |
| 514 | 6CA>5CA | No | D-Loop | Noncoding | Noncoding | Primary PCA | CH-01 | Targeted Seq | Chen et al. 2002 |
| 557 | C>T | Yes | D-Loop | Noncoding | Noncoding | Primary PCA | M38 | Whole mtDNA | McCrow et al. 2015 |
| 567 | A>AC | Yes | D-Loop | Noncoding | Noncoding | Bone Metastasis | SM177 | Whole mtDNA | Hovens et al. 2017 |
| 605 | T>C | Yes | tRNA-Phe | Noncoding | tRNA | Primary PCA | JU-195 | Whole mtDNA | Ju et al. 2014 |
| 709 | G>A | Yes | RNR1 | Noncoding | rRNA | Primary PCA | KB-PCA020 | Whole mtDNA | Kloss-Brandstatter et al. 2010 |
| 709 | G>A | Yes | RNR1 | Noncoding | rRNA | Primary PCA | M18 | Whole mtDNA | McCrow et al. 2015 |
| 723 | A>G | Yes | RNR1 | Noncoding | rRNA | Primary PCA | JU-180 | Whole mtDNA | Ju et al. 2014 |
| 762 | G>A | Yes | RNR1 | Noncoding | rRNA | Primary PCA | M35 | Whole mtDNA | McCrow et al. 2015 |
| 879 | T>C | Yes | RNR1 | Noncoding | rRNA | Primary PCA | KB-PCA012 | Whole mtDNA | Kloss-Brandstatter et al. 2010 |
| 901 | G>A | Yes | RNR1 | Noncoding | rRNA | Primary PCA | AK-11919 | Whole mtDNA | Kalsbeek et al. 2016 |
| 902 | G>A | Yes | RNR1 | Noncoding | rRNA | Primary PCA | JU-1155 | Whole mtDNA | Ju et al. 2014 |
| 902 | G>A | Yes | RNR1 | Noncoding | rRNA | Primary PCA | M27 | Whole mtDNA | McCrow et al. 2015 |
| 943 | G>A | Yes | RNR1 | Noncoding | rRNA | Primary PCA | AK-12596 | Whole mtDNA | Kalsbeek et al. 2016 |
| 955 | AC>A | Yes | RNR1 | Noncoding | Noncoding | Primary PCA | JU-45 | Whole mtDNA | Ju et al. 2014 |
| 965 | 4C>5C | Yes | RNR1 | Noncoding | rRNA | Primary PCA | KB-PCA006 | Whole mtDNA | Kloss-Brandstatter et al. 2010 |
| 992 | T>C | Yes | RNR1 | Noncoding | rRNA | Primary PCA | JU-179 | Whole mtDNA | Ju et al. 2014 |
| 1000 | C>T | No | RNR1 | Noncoding | rRNA | Primary PCA | AK-13034 | Whole mtDNA | Kalsbeek et al. 2016 |
| 1028 | G>A | Yes | RNR1 | Noncoding | rRNA | Primary PCA | JU-174 | Whole mtDNA | Ju et al. 2014 |
| 1169 | G>A | Yes | RNR1 | Noncoding | rRNA | Primary PCA | AK-17212 | Whole mtDNA | Kalsbeek et al. 2016 |
| 1175 | G>A | Yes | RNR1 | Noncoding | rRNA | Primary PCA | JU-180 | Whole mtDNA | Ju et al. 2014 |
| 1180 | T>C | Yes | RNR1 | Noncoding | Noncoding | BPH | Pedi | Whole mtDNA | McCrow et al. 2015 |
| 1207 | T>C | Yes | RNR1 | Noncoding | rRNA | Primary PCA | JU-153 | Whole mtDNA | Ju et al. 2014 |
| 1210 | T>C | Yes | RNR1 | Noncoding | Noncoding | BPH | Ndebele | Whole mtDNA | McCrow et al. 2015 |
| 1249 | T>C | Yes | RNR1 | Noncoding | rRNA | Primary PCA | JU-154 | Whole mtDNA | Ju et al. 2014 |
| 1282 | G>A | Yes | RNR1 | Noncoding | rRNA | Primary PCA | AK-5684 | Whole mtDNA | Kalsbeek et al. 2016 |
| 1301 | G>A | Yes | RNR1 | Noncoding | Noncoding | Bone Metastasis | SM002 | Whole mtDNA | Hovens et al. 2017 |
| 1328 | G>A | Yes | RNR1 | Noncoding | rRNA | Primary PCA | M22 | Whole mtDNA | McCrow et al. 2015 |
| 1339 | G>A | Yes | RNR1 | Noncoding | rRNA | Primary PCA | AK-5828 | Whole mtDNA | Kalsbeek et al. 2016 |
| 1339 | G>A | Yes | RNR1 | Noncoding | rRNA | Primary PCA | JU-176 | Whole mtDNA | Ju et al. 2014 |
| 1350 | G>A | Yes | RNR1 | Noncoding | rRNA | Primary PCA | JU-134 | Whole mtDNA | Ju et al. 2014 |
| 1351 | G>A | Yes | RNR1 | Noncoding | rRNA | Primary PCA | AK-5867 | Whole mtDNA | Kalsbeek et al. 2016 |
| 1410 | G>A | Yes | RNR1 | Noncoding | rRNA | Primary PCA | M43 | Whole mtDNA | McCrow et al. 2015 |
| 1411 | G>A | Yes | RNR1 | Noncoding | rRNA | Primary PCA | M29 | Whole mtDNA | McCrow et al. 2015 |
| 1415 | G>A | Yes | RNR1 | Noncoding | rRNA | Primary PCA | JU-154 | Whole mtDNA | Ju et al. 2014 |
| 1426 | T>C | Yes | RNR1 | Noncoding | rRNA | Primary PCA | AK-5339 | Whole mtDNA | Kalsbeek et al. 2016 |
| 1428 | G>A | Yes | RNR1 | Noncoding | rRNA | Primary PCA | JU-132 | Whole mtDNA | Ju et al. 2014 |
| 1447 | G>A | Yes | RNR1 | Noncoding | rRNA | Primary PCA | M29 | Whole mtDNA | McCrow et al. 2015 |
| 1464 | G>A | Yes | RNR1 | Noncoding | rRNA | Primary PCA | AK-11114 | Whole mtDNA | Kalsbeek et al. 2016 |
| 1464 | G>A | Yes | RNR1 | Noncoding | Noncoding | Soft Tissue Metastasis | AR-214 | Whole mtDNA | Arnold et al. 2015 |
| 1485 | G>A | Yes | RNR1 | Noncoding | rRNA | Primary PCA | M55 | Whole mtDNA | McCrow et al. 2015 |
| 1549 | G>A | Yes | RNR1 | Noncoding | rRNA | Primary PCA | JU-188 | Whole mtDNA | Ju et al. 2014 |
| 1552 | G>A | Yes | RNR1 | Noncoding | rRNA | Primary PCA | JU-94 | Whole mtDNA | Ju et al. 2014 |
| 1623 | A>G | Yes | tRNA-Val | Noncoding | tRNA | Primary PCA | KB-PCA017 | Whole mtDNA | Kloss-Brandstatter et al. 2010 |
| 1660 | G>A | Yes | tRNA-Val | Noncoding | tRNA | Primary PCA | JU-201 | Whole mtDNA | Ju et al. 2014 |
| 1730 | T>C | Yes | RNR2 | Noncoding | rRNA | Primary PCA | JU-191 | Whole mtDNA | Ju et al. 2014 |
| 1748 | G>A | Yes | RNR2 | Noncoding | Noncoding | BPH | Coloured | Whole mtDNA | McCrow et al. 2015 |
| 1750 | G>A | Yes | RNR2 | Noncoding | rRNA | Primary PCA | M38 | Whole mtDNA | McCrow et al. 2015 |
| 1766 | T>C | Yes | RNR2 | Noncoding | rRNA | Primary PCA | M20 | Whole mtDNA | McCrow et al. 2015 |
| 1767 | G>A | Yes | RNR2 | Noncoding | rRNA | Primary PCA | AK-5401 | Whole mtDNA | Kalsbeek et al. 2016 |
| 1770 | G>A | Yes | RNR2 | Noncoding | rRNA | Primary PCA | M54 | Whole mtDNA | McCrow et al. 2015 |
| 1792 | G>A | No | RNR2 | Noncoding | rRNA | Primary PCA | GZ-09 | Targeted Seq | Gomez-Zaera et al. 2006 |
| 1806 | T>C | Yes | RNR2 | Noncoding | rRNA | Primary PCA | JU-825 | Whole mtDNA | Ju et al. 2014 |
| 1806 | T>C | Yes | RNR2 | Noncoding | rRNA | Primary PCA | JU-826 | Whole mtDNA | Ju et al. 2014 |
| 1914 | A>G | Yes | RNR2 | Noncoding | rRNA | Primary PCA | M15 | Whole mtDNA | McCrow et al. 2015 |
| 1988 | G>A | Yes | RNR2 | Noncoding | rRNA | Primary PCA | AK-13343 | Whole mtDNA | Kalsbeek et al. 2016 |
| 2007 | T>C | Yes | RNR2 | Noncoding | rRNA | Primary PCA | KB-PCA003 | Whole mtDNA | Kloss-Brandstatter et al. 2010 |
| 2054 | T>C | NA | RNR2 | Noncoding | Noncoding | Primary PCA | IC-DO36362 | Whole mtDNA | ICGC et al. 2016 |
| 2055 | T>C | Yes | RNR2 | Noncoding | rRNA | Primary PCA | M41 | Whole mtDNA | McCrow et al. 2015 |
| 2119 | 3T>4T | Yes | RNR2 | Noncoding | rRNA | Primary PCA | KB-PCA012 | Whole mtDNA | Kloss-Brandstatter et al. 2010 |
| 2150 | T>del | Yes | RNR2 | Noncoding | rRNA | Primary PCA | KB-PCA005 | Whole mtDNA | Kloss-Brandstatter et al. 2010 |
| 2151 | A>del | Yes | RNR2 | Noncoding | rRNA | Primary PCA | KB-PCA005 | Whole mtDNA | Kloss-Brandstatter et al. 2010 |
| 2205 | T>C | Yes | RNR2 | Noncoding | Noncoding | Primary PCA | IC-DO36343 | Whole mtDNA | ICGC et al. 2016 |
| 2269 | G>A | Yes | RNR2 | Noncoding | rRNA | Primary PCA | M27 | Whole mtDNA | McCrow et al. 2015 |
| 2300 | G>A | Yes | RNR2 | Noncoding | rRNA | Primary PCA | M49 | Whole mtDNA | McCrow et al. 2015 |
| 2302 | T>C | NA | RNR2 | Noncoding | Noncoding | Primary PCA | IC-DO36362 | Whole mtDNA | ICGC et al. 2016 |
| 2304 | G>A | Yes | RNR2 | Noncoding | rRNA | Primary PCA | M13 | Whole mtDNA | McCrow et al. 2015 |
| 2389 | C>T | Yes | RNR2 | Noncoding | Noncoding | Bone Metastasis | AR-140 | Whole mtDNA | Arnold et al. 2015 |
| 2408 | T>C | Yes | RNR2 | Noncoding | rRNA | Primary PCA | JU-178 | Whole mtDNA | Ju et al. 2014 |
| 2425 | A>G | Yes | RNR2 | Noncoding | rRNA | Primary PCA | JU-175 | Whole mtDNA | Ju et al. 2014 |
| 2440 | G>A | Yes | RNR2 | Noncoding | Noncoding | Bone Metastasis | SM001 | Whole mtDNA | Hovens et al. 2017 |
| 2470 | G>A | Yes | RNR2 | Noncoding | rRNA | Primary PCA | M44 | Whole mtDNA | McCrow et al. 2015 |
| 2478 | G>A | Yes | RNR2 | Noncoding | rRNA | Primary PCA | M31 | Whole mtDNA | McCrow et al. 2015 |
| 2492 | G>A | Yes | RNR2 | Noncoding | rRNA | Primary PCA | M50 | Whole mtDNA | McCrow et al. 2015 |
| 2537 | G>A | Yes | RNR2 | Noncoding | rRNA | Primary PCA | JU-196 | Whole mtDNA | Ju et al. 2014 |
| 2545 | T>C | Yes | RNR2 | Noncoding | rRNA | Primary PCA | KB-PCA003 | Whole mtDNA | Kloss-Brandstatter et al. 2010 |
| 2602 | T>C | Yes | RNR2 | Noncoding | rRNA | Primary PCA | JU-48 | Whole mtDNA | Ju et al. 2014 |
| 2622 | G>A | Yes | RNR2 | Noncoding | Noncoding | Primary PCA | IC-DO36308 | Whole mtDNA | ICGC et al. 2016 |
| 2636 | G>A | Yes | RNR2 | Noncoding | rRNA | Primary PCA | AK-12604 | Whole mtDNA | Kalsbeek et al. 2016 |
| 2646 | G>A | Yes | RNR2 | Noncoding | rRNA | Primary PCA | M20 | Whole mtDNA | McCrow et al. 2015 |
| 2652 | G>A | Yes | RNR2 | Noncoding | rRNA | Primary PCA | M43 | Whole mtDNA | McCrow et al. 2015 |
| 2656 | T>C | Yes | RNR2 | Noncoding | rRNA | Primary PCA | JU-45 | Whole mtDNA | Ju et al. 2014 |
| 2673 | G>A | Yes | RNR2 | Noncoding | rRNA | Primary PCA | M41 | Whole mtDNA | McCrow et al. 2015 |
| 2706 | A>G | No | RNR2 | Noncoding | Noncoding | Bone Metastasis | AR-140 | Whole mtDNA | Arnold et al. 2015 |
| 2721 | G>A | Yes | RNR2 | Noncoding | rRNA | Primary PCA | JU-173 | Whole mtDNA | Ju et al. 2014 |
| 2732 | G>A | Yes | RNR2 | Noncoding | rRNA | Primary PCA | JU-42 | Whole mtDNA | Ju et al. 2014 |
| 2814 | G>C | Yes | RNR2 | Noncoding | rRNA | Primary PCA | M44 | Whole mtDNA | McCrow et al. 2015 |
| 2816 | G>A | Yes | RNR2 | Noncoding | rRNA | Primary PCA | JU-127 | Whole mtDNA | Ju et al. 2014 |
| 2817 | G>A | Yes | RNR2 | Noncoding | Noncoding | Primary PCA | IC-DO36248 | Whole mtDNA | ICGC et al. 2016 |
| 2819 | G>A | Yes | RNR2 | Noncoding | rRNA | Primary PCA | JU-188 | Whole mtDNA | Ju et al. 2014 |
| 2819 | G>del ACCTCGGAGCAGAACCCA | Yes | RNR2 | Noncoding | Noncoding | Primary PCA | JU-45 | Whole mtDNA | Ju et al. 2014 |
| 2819 | G>A | Yes | RNR2 | Noncoding | rRNA | Primary PCA | M56 | Whole mtDNA | McCrow et al. 2015 |
| 2828 | G>A | Yes | RNR2 | Noncoding | rRNA | Primary PCA | AK-5958 | Whole mtDNA | Kalsbeek et al. 2016 |
| 2852 | C>T | Yes | RNR2 | Noncoding | rRNA | Primary PCA | JU-200 | Whole mtDNA | Ju et al. 2014 |
| 2876 | G>A | Yes | RNR2 | Noncoding | rRNA | Primary PCA | JU-180 | Whole mtDNA | Ju et al. 2014 |
| 2896 | G>A | Yes | RNR2 | Noncoding | rRNA | Primary PCA | JU-1364 | Whole mtDNA | Ju et al. 2014 |
| 2909 | G>A | Yes | RNR2 | Noncoding | rRNA | Primary PCA | JU-45 | Whole mtDNA | Ju et al. 2014 |
| 2916 | G>A | Yes | RNR2 | Noncoding | Noncoding | Primary PCA | IC-DO36351 | Whole mtDNA | ICGC et al. 2016 |
| 2923 | G>A | No | RNR2 | Noncoding | rRNA | Primary PCA | JE-32 | Targeted Seq | Jeronimo et al. 2001 |
| 2932 | G>A | Yes | RNR2 | Noncoding | rRNA | Primary PCA | JU-144 | Whole mtDNA | Ju et al. 2014 |
| 2943 | G>A | Yes | RNR2 | Noncoding | rRNA | Primary PCA | M44 | Whole mtDNA | McCrow et al. 2015 |
| 3004 | C>T | Yes | RNR2 | Noncoding | rRNA | Primary PCA | M53 | Whole mtDNA | McCrow et al. 2015 |
| 3031 | G>A | No | RNR2 | Noncoding | Noncoding | Primary PCA | IC-DO36292 | Whole mtDNA | ICGC et al. 2016 |
| 3036 | G>A | Yes | RNR2 | Noncoding | rRNA | Primary PCA | AK-11538 | Whole mtDNA | Kalsbeek et al. 2016 |
| 3047 | G>A | Yes | RNR2 | Noncoding | rRNA | Primary PCA | JU-148 | Whole mtDNA | Ju et al. 2014 |
| 3061 | G>A | Yes | RNR2 | Noncoding | Noncoding | BPH | Ndebele | Whole mtDNA | McCrow et al. 2015 |
| 3061 | G>A | Yes | RNR2 | Noncoding | Noncoding | Primary PCA | IC-DO36305 | Whole mtDNA | ICGC et al. 2016 |
| 3063 | G>A | Yes | RNR2 | Noncoding | Noncoding | Primary PCA | IC-DO36294 | Whole mtDNA | ICGC et al. 2016 |
| 3068 | G>A | Yes | RNR2 | Noncoding | rRNA | Primary PCA | M48 | Whole mtDNA | McCrow et al. 2015 |
| 3070 | G>A | Yes | RNR2 | Noncoding | rRNA | Primary PCA | JU-185 | Whole mtDNA | Ju et al. 2014 |
| 3079 | G>A | Yes | RNR2 | Noncoding | rRNA | Primary PCA | JU-184 | Whole mtDNA | Ju et al. 2014 |
| 3080 | G>A | Yes | RNR2 | Noncoding | rRNA | Primary PCA | JU-173 | Whole mtDNA | Ju et al. 2014 |
| 3098 | T>C | Yes | RNR2 | Noncoding | Noncoding | Bone Metastasis | SM498 | Whole mtDNA | Hovens et al. 2017 |
| 3173 | G>A | Yes | RNR2 | Noncoding | rRNA | Primary PCA | M29 | Whole mtDNA | McCrow et al. 2015 |
| 3193 | T>C | Yes | RNR2 | Noncoding | rRNA | Primary PCA | M21 | Whole mtDNA | McCrow et al. 2015 |
| 3255 | G>A | Yes | tRNA-Leu1 | Noncoding | tRNA | Primary PCA | AK-4691 | Whole mtDNA | Kalsbeek et al. 2016 |
| 3308 | NA | NA | ND1 | NA | NA | Primary PCA | NA | Whole mtDNA | Parr et al. 2006 |
| 3350 | T>C | Yes | ND1 | I15T | Benign | Primary PCA | LIND-SWE-38 | Whole mtDNA | Lindberg et al. 2013 |
| 3357 | G>A | Yes | ND1 | M17M | Synonymous SNV | Primary PCA | M40 | Whole mtDNA | McCrow et al. 2015 |
| 3357 | G>A | No | ND1 | M17M | Synonymous SNV | Primary PCA | JE-01 | Targeted Seq | Jeronimo et al. 2001 |
| 3380 | G>A | Yes | ND1 | R25Q | Probably damaging | Primary PCA | JU-1286 | Whole mtDNA | Ju et al. 2014 |
| 3394 | T>C | Yes | ND1 | Y30H | Benign | Primary PCA | KB-PCA013 | Whole mtDNA | Kloss-Brandstatter et al. 2010 |
| 3394 | NA | NA | ND1 | NA | NA | Primary PCA | NA | Whole mtDNA | Parr et al. 2006 |
| 3398 | NA | NA | ND1 | NA | NA | Primary PCA | NA | Whole mtDNA | Parr et al. 2006 |
| 3428 | G>A | Yes | ND1 | G41D | Probably damaging | Primary PCA | LIND-SWE-1 | Whole mtDNA | Lindberg et al. 2013 |
| 3434 | A>G | No | ND1 | Y43C | Benign | Primary PCA | JE-01 | Targeted Seq | Jeronimo et al. 2001 |
| 3438 | G>A | Yes | ND1 | G44G | Synonymous SNV | Primary PCA | JU-179 | Whole mtDNA | Ju et al. 2014 |
| 3441 | G>A | No | ND1 | L45L | Synonymous SNV | Bone Metastasis | AR-050 | Whole mtDNA | Arnold et al. 2015 |
| 3454 | G>A | Yes | ND1 | A50T | Probably damaging | Primary PCA | JU-186 | Whole mtDNA | Ju et al. 2014 |
| 3454 | G>A | Yes | ND1 | A50T | Probably damaging | Primary PCA | M47 | Whole mtDNA | McCrow et al. 2015 |
| 3480 | A>G | No | ND1 | K58K | Synonymous SNV | Primary PCA | JE-01 | Targeted Seq | Jeronimo et al. 2001 |
| 3492 | A>C | Yes | ND1 | K62N | Benign | Bone Metastasis | SM001 | Whole mtDNA | Hovens et al. 2017 |
| 3505 | A>G | No | ND1 | T67A | Benign | Primary PCA | JE-01 | Targeted Seq | Jeronimo et al. 2001 |
| 3526 | G>A | Yes | ND1 | G557A | Benign | Primary PCA | IC-DO36248 | Whole mtDNA | ICGC et al. 2016 |
| 3531 | G>A | Yes | ND1 | P75P | Synonymous SNV | Primary PCA | JU-1286 | Whole mtDNA | Ju et al. 2014 |
| 3565 | A>AC | Yes | ND1 | Frameshift | Frameshift | Primary PCA | JU-45 | Whole mtDNA | Ju et al. 2014 |
| 3578 | T>C | Yes | ND1 | M91T | Possibly damaging | Primary PCA | JU-49 | Whole mtDNA | Ju et al. 2014 |
| 3664 | G>A | Yes | ND1 | G120X | Stopgain | Primary PCA | JU-825 | Whole mtDNA | Ju et al. 2014 |
| 3664 | G>A | Yes | ND1 | G120X | Stopgain | Primary PCA | JU-826 | Whole mtDNA | Ju et al. 2014 |
| 3720 | A>G | No | ND1 | Q138Q | Noncoding | Primary PCA | AR-380 | Whole mtDNA | Arnold et al. 2015 |
| 3727 | T>C | Yes | ND1 | S141P | Probably damaging | Primary PCA | AK-14732 | Whole mtDNA | Kalsbeek et al. 2016 |
| 3793 | T>C | Yes | ND1 | S163P | Possibly damaging | Primary PCA | JU-146 | Whole mtDNA | Ju et al. 2014 |
| 3795 | C>T | Yes | ND1 | S163S | Synonymous SNV | Primary PCA | M20 | Whole mtDNA | McCrow et al. 2015 |
| 3834 | G>A | Yes | ND1 | L176L | Synonymous SNV | Primary PCA | AK-5339 | Whole mtDNA | Kalsbeek et al. 2016 |
| 3915 | G>A | Yes | ND1 | G203G | Synonymous SNV | Primary PCA | AK-6087 | Whole mtDNA | Kalsbeek et al. 2016 |
| 3915 | G>A | Yes | ND1 | G203G | Synonymous SNV | Primary PCA | LIND-SWE-26 | Whole mtDNA | Lindberg et al. 2013 |
| 3935 | G>A | Yes | ND1 | G210D | Probably damaging | Primary PCA | JU-195 | Whole mtDNA | Ju et al. 2014 |
| 4007 | T>C | Yes | ND1 | M234T | Probably damaging | Primary PCA | JU-144 | Whole mtDNA | Ju et al. 2014 |
| 4057 | T>C | Yes | ND1 | S251P | Benign | Primary PCA | JU-188 | Whole mtDNA | Ju et al. 2014 |
| 4086 | C>T | Yes | ND1 | V260V | Synonymous SNV | Primary PCA | M51 | Whole mtDNA | McCrow et al. 2015 |
| 4216 | T>C | Yes | ND1 | Y304H | benign | Soft Tissue Metastasis | AR-069 | Whole mtDNA | Arnold et al. 2015 |
| 4216 | NA | NA | ND1 | NA | NA | Primary PCA | NA | Whole mtDNA | Parr et al. 2006 |
| 4216 | C>T | No | ND1 | H340Y | Benign | Bone Metastasis | AR-380 | Whole mtDNA | Arnold et al. 2015 |
| 4217 | NA | NA | ND1 | NA | NA | Primary PCA | NA | Whole mtDNA | Parr et al. 2006 |
| 4309 | G>A | Yes | tRNA-Ile | Noncoding | tRNA | Primary PCA | AK-13179 | Whole mtDNA | Kalsbeek et al. 2016 |
| 4350 | C>T | Yes | tRNA-Gln | Noncoding | tRNA | Primary PCA | JU-44 | Whole mtDNA | Ju et al. 2014 |
| 4408 | G>A | Yes | tRNA-Met | Noncoding | tRNA | Primary PCA | JU-129 | Whole mtDNA | Ju et al. 2014 |
| 4412 | G>A | Yes | tRNA-Met | Noncoding | tRNA | Primary PCA | AK-5288 | Whole mtDNA | Kalsbeek et al. 2016 |
| 4475 | T>C | Yes | ND2 | N2N | Synonymous SNV | Primary PCA | LIND-SWE-38 | Whole mtDNA | Lindberg et al. 2013 |
| 4481 | G>A | Yes | ND2 | L4L | Synonymous SNV | Primary PCA | LIND-SWE-1 | Whole mtDNA | Lindberg et al. 2013 |
| 4522 | T>C | Yes | ND2 | L18P | Probably damaging | Primary PCA | AK-5874 | Whole mtDNA | Kalsbeek et al. 2016 |
| 4522 | T>C | Yes | ND2 | L18P | Probably damaging | Primary PCA | KB-PCA012 | Whole mtDNA | Kloss-Brandstatter et al. 2010 |
| 4525 | T>C | Yes | ND2 | I19T | Benign | Primary PCA | M47 | Whole mtDNA | McCrow et al. 2015 |
| 4546 | G>A | Yes | ND2 | W26X | Stopgain | Primary PCA | M30 | Whole mtDNA | McCrow et al. 2015 |
| 4561 | T>C | Yes | ND2 | V31A | Benign | Primary PCA | JU-127 | Whole mtDNA | Ju et al. 2014 |
| 4561 | C>T | No | ND2 | A31V | Benign | Soft Tissue Metastasis | AR-069 | Whole mtDNA | Arnold et al. 2015 |
| 4569 | NA | NA | ND2 | NA | NA | Primary PCA | NA | Whole mtDNA | Parr et al. 2006 |
| 4591 | NA | NA | ND2 | NA | NA | Primary PCA | NA | Whole mtDNA | Parr et al. 2006 |
| 4716 | NA | NA | ND2 | NA | NA | Primary PCA | NA | Whole mtDNA | Parr et al. 2006 |
| 4720 | G>A | Yes | ND2 | W84X | Stopgain | Primary PCA | JU-197 | Whole mtDNA | Ju et al. 2014 |
| 4722 | NA | NA | ND2 | NA | NA | Primary PCA | NA | Whole mtDNA | Parr et al. 2006 |
| 4769 | A>G | Yes | ND2 | M100M | Synonymous SNV | Soft Tissue Metastasis | AR-050 | Whole mtDNA | Arnold et al. 2015 |
| 4812 | G>A | Yes | ND2 | V115I | Benign | Primary PCA | JU-196 | Whole mtDNA | Ju et al. 2014 |
| 4861 | T>C | Yes | ND2 | L131P | Probably damaging | Primary PCA | M44 | Whole mtDNA | McCrow et al. 2015 |
| 4864 | NA | NA | ND2 | NA | NA | Primary PCA | NA | Whole mtDNA | Parr et al. 2006 |
| 4917 | A>G | Yes | ND2 | N150D | Benign | Primary PCA | LIND-SWE-13 | Whole mtDNA | Lindberg et al. 2013 |
| 4917 | NA | NA | ND2 | NA | NA | Primary PCA | NA | Whole mtDNA | Parr et al. 2006 |
| 4969 | G>A | Yes | ND2 | W167X | Stopgain | Primary PCA | JU-200 | Whole mtDNA | Ju et al. 2014 |
| 5031 | A>G | Yes | ND2 | G188G | Synonymous SNV | Primary PCA | KB-PCA022 | Whole mtDNA | Kloss-Brandstatter et al. 2010 |
| 5043 | G>A | Yes | ND2 | A192T | Benign | Primary PCA | LIND-SWE-14 | Whole mtDNA | Lindberg et al. 2013 |
| 5043 | G>A | Yes | ND2 | A192T | Benign | Primary PCA | LIND-SWE-26 | Whole mtDNA | Lindberg et al. 2013 |
| 5046 | G>A | Yes | ND2 | V193I | Benign | Primary PCA | LIND-SWE-37 | Whole mtDNA | Lindberg et al. 2013 |
| 5105 | T>C | Yes | ND2 | T212T | Synonymous SNV | Primary PCA | JU-200 | Whole mtDNA | Ju et al. 2014 |
| 5140 | G>A | Yes | ND2 | S224N | Benign | Primary PCA | AK-12008 | Whole mtDNA | Kalsbeek et al. 2016 |
| 5147 | G>A | Yes | ND2 | T226T | Synonymous SNV | Primary PCA | M16 | Whole mtDNA | McCrow et al. 2015 |
| 5177 | G>A | Yes | ND2 | K236K | Synonymous SNV | Primary PCA | LIND-SWE-52 | Whole mtDNA | Lindberg et al. 2013 |
| 5237 | G>A | Yes | ND2 | P256P | Synonymous SNV | Primary PCA | M55 | Whole mtDNA | McCrow et al. 2015 |
| 5277 | T>C | Yes | ND2 | F270L | Benign | Primary PCA | LIND-SWE-44 | Whole mtDNA | Lindberg et al. 2013 |
| 5294 | C>T | Yes | ND2 | S275S | Synonymous SNV | Primary PCA | M11 | Whole mtDNA | McCrow et al. 2015 |
| 5296 | T>C | Yes | ND2 | L276P | Probably damaging | Primary PCA | AK-4691 | Whole mtDNA | Kalsbeek et al. 2016 |
| 5371 | NA | NA | ND2 | NA | NA | Primary PCA | NA | Whole mtDNA | Parr et al. 2006 |
| 5393 | T>C | Yes | ND2 | S308S | Synonymous SNV | Primary PCA | KB-PCA014 | Whole mtDNA | Kloss-Brandstatter et al. 2010 |
| 5424 | NA | NA | ND2 | NA | NA | Primary PCA | NA | Whole mtDNA | Parr et al. 2006 |
| 5460 | G>A | Yes | ND2 | A331T | Benign | Primary PCA | LIND-SWE-45 | Whole mtDNA | Lindberg et al. 2013 |
| 5521 | G>A | Yes | tRNA-Trp | Noncoding | tRNA | Primary PCA | JU-1364 | Whole mtDNA | Ju et al. 2014 |
| 5590 | G>A | Yes | tRNA-Ala | Noncoding | tRNA | Primary PCA | JU-134 | Whole mtDNA | Ju et al. 2014 |
| 5590 | G>A | Yes | tRNA-Ala | Noncoding | tRNA | Primary PCA | JU-197 | Whole mtDNA | Ju et al. 2014 |
| 5593 | NA | NA | tRNA-Ala | Noncoding | tRNA | Primary PCA | NA | Whole mtDNA | Parr et al. 2006 |
| 5650 | NA | NA | tRNA-Ala | Noncoding | tRNA | Primary PCA | NA | Whole mtDNA | Parr et al. 2006 |
| 5655 | NA | NA | tRNA-Ala | Noncoding | tRNA | Primary PCA | NA | Whole mtDNA | Parr et al. 2006 |
| 5663 | NA | NA | tRNA-Ala | Noncoding | tRNA | Primary PCA | NA | Whole mtDNA | Parr et al. 2006 |
| 5677 | NA | NA | tRNA-Asn | Noncoding | tRNA | Primary PCA | NA | Whole mtDNA | Parr et al. 2006 |
| 5779 | AGCT>A | Yes | tRNA-Cys | Noncoding | Noncoding | Primary PCA | JU-94 | Whole mtDNA | Ju et al. 2014 |
| 5782 | T>A | Yes | tRNA-Cys | Noncoding | tRNA | Primary PCA | JU-94 | Whole mtDNA | Ju et al. 2014 |
| 5850 | T>C | Yes | tRNA-Tyr | Noncoding | Noncoding | BPH | Ndebele | Whole mtDNA | McCrow et al. 2015 |
| 5882 | NA | NA | tRNA-Tyr | Noncoding | tRNA | Primary PCA | NA | Whole mtDNA | Parr et al. 2006 |
| 5894 | A>AC | Yes | NC | Noncoding | Noncoding | Primary PCA | JU-148 | Whole mtDNA | Ju et al. 2014 |
| 5894 | AC>A | Yes | NC | Noncoding | Noncoding | Primary PCA | JU-188 | Whole mtDNA | Ju et al. 2014 |
| 5902 | C>T | No | NC | Noncoding | Noncoding | Soft Tissue Metastasis | AR-108 | Whole mtDNA | Arnold et al. 2015 |
| 5949 | G>A | Yes | COX1 | G16X | Stopgain | Primary PCA | PE-p22 | Targeted Seq | Petros et al. 2005 |
| 5979 | G>A | Yes | COX1 | A26T | Probably damaging | Primary PCA | M47 | Whole mtDNA | McCrow et al. 2015 |
| 6009 | NA | NA | COX1 | NA | NA | Primary PCA | NA | Whole mtDNA | Parr et al. 2006 |
| 6016 | G>A | Yes | COX1 | R38Q | Probably damaging | Primary PCA | LIND-SWE-8 | Whole mtDNA | Lindberg et al. 2013 |
| 6021 | G>A | Yes | COX1 | E40K | Probably damaging | Primary PCA | M19 | Whole mtDNA | McCrow et al. 2015 |
| 6037 | NA | NA | COX1 | NA | NA | Primary PCA | NA | Whole mtDNA | Parr et al. 2006 |
| 6109 | T>C | Yes | COX1 | M69T | Benign | Primary PCA | M43 | Whole mtDNA | McCrow et al. 2015 |
| 6174 | G>A | Yes | COX1 | D91N | Probably damaging | Primary PCA | M43 | Whole mtDNA | McCrow et al. 2015 |
| 6178 | T>C | Yes | COX1 | M92T | Probably damaging | Primary PCA | AK-5785 | Whole mtDNA | Kalsbeek et al. 2016 |
| 6211 | G>A | Yes | COX1 | W103X | Stopgain | Primary PCA | M20 | Whole mtDNA | McCrow et al. 2015 |
| 6219 | NA | NA | COX1 | NA | NA | Primary PCA | NA | Whole mtDNA | Parr et al. 2006 |
| 6277 | G>A | Yes | COX1 | G125D | Probably damaging | Primary PCA | JU-1363 | Whole mtDNA | Ju et al. 2014 |
| 6284 | G>A | No | COX1 | T127T | Synonymous SNV | Primary PCA | AK-5405 | Whole mtDNA | Kalsbeek et al. 2016 |
| 6303 | G>A | Yes | COX1 | G134X | Stopgain | Primary PCA | M27 | Whole mtDNA | McCrow et al. 2015 |
| 6307 | NA | NA | COX1 | NA | NA | Primary PCA | NA | Whole mtDNA | Parr et al. 2006 |
| 6312 | T>C | Yes | COX1 | S137P | Possibly damaging | Primary PCA | JU-1156 | Whole mtDNA | Ju et al. 2014 |
| 6345 | T>C | Yes | COX1 | F148L | Probably damaging | Primary PCA | M36 | Whole mtDNA | McCrow et al. 2015 |
| 6380 | A>del | Yes | COX1 | Frameshift | Frameshift | Primary PCA | LIND-SWE-5 | Whole mtDNA | Lindberg et al. 2013 |
| 6382 | NA | NA | COX1 | NA | NA | Primary PCA | NA | Whole mtDNA | Parr et al. 2006 |
| 6384 | G>A | Yes | COX1 | A161T | Probably damaging | Primary PCA | KB-PCA007 | Whole mtDNA | Kloss-Brandstatter et al. 2010 |
| 6384 | G>A | Yes | COX1 | A161T | Probably damaging | Primary PCA | M18 | Whole mtDNA | McCrow et al. 2015 |
| 6456 | G>A | Yes | COX1 | V185I | Benign | Primary PCA | JU-188 | Whole mtDNA | Ju et al. 2014 |
| 6460 | G>A | Yes | COX1 | W186X | Stopgain | Primary PCA | IC-DO36255 | Whole mtDNA | ICGC et al. 2016 |
| 6510 | G>A | Yes | COX1 | A203T | Probably damaging | Primary PCA | JU-142 | Whole mtDNA | Ju et al. 2014 |
| 6535 | C>T | Yes | COX1 | T211M | Probably damaging | Primary PCA | LIND-SWE-8 | Whole mtDNA | Lindberg et al. 2013 |
| 6541 | G>A | Yes | COX1 | R213H | Probably damaging | Primary PCA | AK-5401 | Whole mtDNA | Kalsbeek et al. 2016 |
| 6579 | NA | NA | COX1 | NA | NA | Primary PCA | NA | Whole mtDNA | Parr et al. 2006 |
| 6620 | T>C | Yes | COX1 | G239G | Synonymous SNV | Bone Metastasis | SM177 | Whole mtDNA | Hovens et al. 2017 |
| 6627 | G>A | Yes | COX1 | E242K | Benign | Primary PCA | M38 | Whole mtDNA | McCrow et al. 2015 |
| 6644 | C>T | Yes | COX1 | I247I | Synonymous SNV | Primary PCA | M55 | Whole mtDNA | McCrow et al. 2015 |
| 6691 | NA | NA | COX1 | NA | NA | Primary PCA | NA | Whole mtDNA | Parr et al. 2006 |
| 6709 | G>A | Yes | COX1 | G1123A | Probably damaging | Primary PCA | IC-DO36309 | Whole mtDNA | ICGC et al. 2016 |
| 6718 | G>A | Yes | COX1 | G272D | Probably damaging | Primary PCA | JU-185 | Whole mtDNA | Ju et al. 2014 |
| 6736 | T>C | Yes | COX1 | M278T | Benign | Bone Metastasis | SM002 | Whole mtDNA | Hovens et al. 2017 |
| 6762 | G>A | Yes | COX1 | V287M | Probably damaging | Primary PCA | M40 | Whole mtDNA | McCrow et al. 2015 |
| 6810 | G>A | Yes | COX1 | A303T | Probably damaging | Primary PCA | M19 | Whole mtDNA | McCrow et al. 2015 |
| 6844 | T>C | Yes | COX1 | I314T | Probably damaging | Primary PCA | JU-133 | Whole mtDNA | Ju et al. 2014 |
| 6852 | G>A | Yes | COX1 | G317S | Probably damaging | Primary PCA | JU-135 | Whole mtDNA | Ju et al. 2014 |
| 6912 | G>A | Yes | COX1 | A337T | Probably damaging | Primary PCA | JU-1156 | Whole mtDNA | Ju et al. 2014 |
| 6930 | G>A | Yes | COX1 | G343X | Stopgain | Primary PCA | AK-5980 | Whole mtDNA | Kalsbeek et al. 2016 |
| 6930 | G>A | Yes | COX1 | G343X | Stopgain | Primary PCA | JU-134 | Whole mtDNA | Ju et al. 2014 |
| 6945 | T>C | Yes | COX1 | F348L | Probably damaging | Primary PCA | JU-193 | Whole mtDNA | Ju et al. 2014 |
| 6955 | G>A | Yes | COX1 | G351D | Probably damaging | Primary PCA | M45 | Whole mtDNA | McCrow et al. 2015 |
| 7013 | G>A | Yes | COX1 | T70T | Synonymous SNV | Primary PCA | LIND-SWE-31 | Whole mtDNA | Lindberg et al. 2013 |
| 7026 | G>A | Yes | COX1 | A375T | Probably damaging | BPH | Pedi | Whole mtDNA | McCrow et al. 2015 |
| 7026 | G>A | Yes | COX1 | A375T | Probably damaging | Primary PCA | IC-DO36349 | Whole mtDNA | ICGC et al. 2016 |
| 7028 | C>T | Yes | COX1 | A375A | Synonymous SNV | Bone Metastasis | AR-069 | Whole mtDNA | Arnold et al. 2015 |
| 7028 | C>T | Yes | COX1 | A375A | Synonymous SNV | Bone Metastasis | AR-140 | Whole mtDNA | Arnold et al. 2015 |
| 7028 | C>T | Yes | COX1 | A375A | Synonymous SNV | Primary PCA | AR-069 | Whole mtDNA | Arnold et al. 2015 |
| 7028 | C>T | Yes | COX1 | A375A | Synonymous SNV | Soft Tissue Metastasis | AR-069 | Whole mtDNA | Arnold et al. 2015 |
| 7059 | NA | NA | COX1 | NA | NA | Primary PCA | NA | Whole mtDNA | Parr et al. 2006 |
| 7072 | T>C | Yes | COX1 | M390T | Probably damaging | Primary PCA | JU-1286 | Whole mtDNA | Ju et al. 2014 |
| 7075 | G>A | Yes | COX1 | G391E | Probably damaging | Primary PCA | JU-160 | Whole mtDNA | Ju et al. 2014 |
| 7108 | G>A | Yes | COX1 | G402D | Probably damaging | Primary PCA | M25 | Whole mtDNA | McCrow et al. 2015 |
| 7131 | G>A | Yes | COX1 | A410T | Benign | Primary PCA | M31 | Whole mtDNA | McCrow et al. 2015 |
| 7140 | C>T | Yes | COX1 | H413Y | Possibly damaging | Primary PCA | JU-130 | Whole mtDNA | Ju et al. 2014 |
| 7146 | NA | NA | COX1 | NA | NA | Primary PCA | NA | Whole mtDNA | Parr et al. 2006 |
| 7159 | NA | NA | COX1 | NA | NA | Primary PCA | NA | Whole mtDNA | Parr et al. 2006 |
| 7194 | C>T | Yes | COX1 | L431F | Probably damaging | Primary PCA | LIND-SWE-21 | Whole mtDNA | Lindberg et al. 2013 |
| 7290 | A>G | Yes | COX1 | T463A | Benign | Primary PCA | JU-159 | Whole mtDNA | Ju et al. 2014 |
| 7293 | G>A | Yes | COX1 | A464T | Probably damaging | Primary PCA | JU-1155 | Whole mtDNA | Ju et al. 2014 |
| 7293 | G>A | Yes | COX1 | A464T | Probably damaging | Primary PCA | M56 | Whole mtDNA | McCrow et al. 2015 |
| 7309 | NA | NA | COX1 | NA | NA | Primary PCA | NA | Whole mtDNA | Parr et al. 2006 |
| 7380 | G>A | Yes | COX1 | E493K | Probably damaging | Primary PCA | M42 | Whole mtDNA | McCrow et al. 2015 |
| 7389 | NA | NA | COX1 | NA | NA | Primary PCA | NA | Whole mtDNA | Parr et al. 2006 |
| 7407 | NA | NA | COX1 | NA | NA | Primary PCA | NA | Whole mtDNA | Parr et al. 2006 |
| 7471 | 6C>7C | Yes | tRNA-Ser1 | Noncoding | tRNA | Primary PCA | KB-PCA015 | Whole mtDNA | Kloss-Brandstatter et al. 2010 |
| 7498 | G>A | Yes | tRNA-Ser1 | Noncoding | tRNA | Primary PCA | JU-182 | Whole mtDNA | Ju et al. 2014 |
| 7521 | NA | NA | tRNA-Asp | Noncoding | tRNA | Primary PCA | NA | Whole mtDNA | Parr et al. 2006 |
| 7588 | G>A | Yes | COX2 | M1M | Synonymous SNV | Primary PCA | M33 | Whole mtDNA | McCrow et al. 2015 |
| 7741 | T>C | Yes | COX2 | N52N | Synonymous SNV | Primary PCA | LIND-SWE-49 | Whole mtDNA | Lindberg et al. 2013 |
| 7742 | A>G | Yes | COX2 | T53A | Probably damaging | Primary PCA | LIND-SWE-9 | Whole mtDNA | Lindberg et al. 2013 |
| 7744 | T>C | Yes | COX2 | T53T | Synonymous SNV | Primary PCA | JU-199 | Whole mtDNA | Ju et al. 2014 |
| 7830 | G>A | Yes | COX2 | R82H | Probably damaging | Primary PCA | JU-181 | Whole mtDNA | Ju et al. 2014 |
| 7854 | T>C | Yes | COX2 | T276C | Benign | Primary PCA | IC-DO36292 | Whole mtDNA | ICGC et al. 2016 |
| 7854 | T>C | Yes | COX2 | V90A | Benign | Primary PCA | JU-1157 | Whole mtDNA | Ju et al. 2014 |
| 7870 | T>C | Yes | COX2 | L95L | Synonymous SNV | Primary PCA | M32 | Whole mtDNA | McCrow et al. 2015 |
| 7871 | A>C | Yes | COX2 | T96P | Probably damaging | Primary PCA | JU-49 | Whole mtDNA | Ju et al. 2014 |
| 7912 | A>G | No | COX2 | E109E | Synonymous SNV | Primary PCA | AK-13104 | Whole mtDNA | Kalsbeek et al. 2016 |
| 7951 | A>G | Yes | COX2 | M122M | Synonymous SNV | Primary PCA | AK-11538 | Whole mtDNA | Kalsbeek et al. 2016 |
| 7965 | T>C | Yes | COX2 | F127S | Probably damaging | Primary PCA | JU-191 | Whole mtDNA | Ju et al. 2014 |
| 7970 | G>A | Yes | COX2 | E129K | Benign | Primary PCA | M45 | Whole mtDNA | McCrow et al. 2015 |
| 7980 | A>G | Yes | COX2 | D132G | Probably damaging | Primary PCA | JU-94 | Whole mtDNA | Ju et al. 2014 |
| 8024 | G>A | Yes | COX2 | E147K | Benign | Primary PCA | JU-144 | Whole mtDNA | Ju et al. 2014 |
| 8027 | NA | NA | COX2 | NA | NA | Primary PCA | NA | Whole mtDNA | Parr et al. 2006 |
| 8037 | G>A | Yes | COX2 | R151H | Probably damaging | Primary PCA | JU-147 | Whole mtDNA | Ju et al. 2014 |
| 8091 | G>A | Yes | COX2 | G169D | Benign | Primary PCA | JU-186 | Whole mtDNA | Ju et al. 2014 |
| 8133 | NA | NA | COX2 | NA | NA | Primary PCA | NA | Whole mtDNA | Parr et al. 2006 |
| 8159 | T>C | Yes | COX2 | Y192H | Probably damaging | BPH | Zulu | Whole mtDNA | McCrow et al. 2015 |
| 8181 | T>C | Yes | COX2 | I199T | Probably damaging | Primary PCA | LIND-SWE-32 | Whole mtDNA | Lindberg et al. 2013 |
| 8184 | G>A | Yes | COX2 | C200Y | Probably damaging | Primary PCA | KB-PCA015 | Whole mtDNA | Kloss-Brandstatter et al. 2010 |
| 8199 | G>A | Yes | COX2 | S205N | Probably damaging | Primary PCA | JU-174 | Whole mtDNA | Ju et al. 2014 |
| 8251 | G>A | Yes | COX2 | G222G | Synonymous SNV | Primary PCA | JU-155 | Whole mtDNA | Ju et al. 2014 |
| 8269 | A>G | Yes | COX2 | X228X | Synonymous SNV | Primary PCA | AK-14365 | Whole mtDNA | Kalsbeek et al. 2016 |
| 8269 | G>A | Yes | COX2 | X228X | Synonymous SNV | Primary PCA | KB-PCA001 | Whole mtDNA | Kloss-Brandstatter et al. 2010 |
| 8270 | C>CACCCCCTCT | Yes | NC | Noncoding | Noncoding | Bone Metastasis | SM001 | Whole mtDNA | Hovens et al. 2017 |
| 8313 | A>G | Yes | tRNA-Lys | Noncoding | tRNA | Primary PCA | KB-PCA005 | Whole mtDNA | Kloss-Brandstatter et al. 2010 |
| 8412 | T>C | Yes | ATP8 | M16T | Possibly damaging | Primary PCA | LIND-SWE-31 | Whole mtDNA | Lindberg et al. 2013 |
| 8616 | NA | NA | ATP6 | NA | NA | Primary PCA | NA | Whole mtDNA | Parr et al. 2006 |
| 8656 | A>T | Yes | ATP6 | T44S | Benign | Bone Metastasis | SM177 | Whole mtDNA | Hovens et al. 2017 |
| 8697 | G>A | Yes | ATP6 | M57M | Synonymous SNV | Primary PCA | LIND-SWE-10 | Whole mtDNA | Lindberg et al. 2013 |
| 8697 | A>G | No | ATP6 | M57M | Synonymous SNV | Bone Metastasis | AR-380 | Whole mtDNA | Arnold et al. 2015 |
| 8701 | NA | NA | ATP6 | NA | NA | Primary PCA | NA | Whole mtDNA | Parr et al. 2006 |
| 8705 | T>C | Yes | ATP6 | M60T | Benign | Primary PCA | KB-PCA024 | Targeted Seq | Kloss-Brandstatter et al. 2010 |
| 8723 | G>A | Yes | ATP6 | R66Q | Benign | Primary PCA | AK-5132 | Whole mtDNA | Kalsbeek et al. 2016 |
| 8736 | T>C | Yes | ATP6 | L70L | Synonymous SNV | Primary PCA | KB-PCA025 | Whole mtDNA | Kloss-Brandstatter et al. 2010 |
| 8746 | T>C | Yes | ATP6 | S74P | Possibly damaging | Primary PCA | LIND-SWE-47 | Whole mtDNA | Lindberg et al. 2013 |
| 8802 | T>C | Yes | ATP6 | F92F | Synonymous SNV | Primary PCA | IC-DO36271 | Whole mtDNA | ICGC et al. 2016 |
| 8893 | NA | NA | ATP6 | NA | NA | Primary PCA | NA | Whole mtDNA | Parr et al. 2006 |
| 8903 | NA | NA | ATP6 | NA | NA | Primary PCA | NA | Whole mtDNA | Parr et al. 2006 |
| 8932 | C>T | No | ATP6 | P136S | Probably damaging | Primary PCA | PE-p20 | Targeted Seq | Petros et al. 2005 |
| 8939 | T>C | Yes | ATP6 | I138T | Probably damaging | Primary PCA | LIND-SWE-32 | Whole mtDNA | Lindberg et al. 2013 |
| 8969 | G>A | Yes | ATP6 | S148N | Probably damaging | Primary PCA | M32 | Whole mtDNA | McCrow et al. 2015 |
| 8977 | A>C | Yes | ATP6 | I151L | Possibly damaging | BPH | Zulu | Whole mtDNA | McCrow et al. 2015 |
| 9064 | G>C | Yes | ATP6 | A180P | Probably damaging | Primary PCA | M41 | Whole mtDNA | McCrow et al. 2015 |
| 9090 | T>C | Yes | ATP6 | S188S | Synonymous SNV | Primary PCA | M29 | Whole mtDNA | McCrow et al. 2015 |
| 9116 | T>C | Yes | ATP6 | I197T | Benign | Primary PCA | KB-PCA027 | Targeted Seq | Kloss-Brandstatter et al. 2010 |
| 9139 | G>A | Yes | ATP6 | A205T | Probably damaging | Primary PCA | AK-12437 | Whole mtDNA | Kalsbeek et al. 2016 |
| 9145 | G>A | Yes | ATP6 | A207T | Probably damaging | Primary PCA | AK-5339 | Whole mtDNA | Kalsbeek et al. 2016 |
| 9188 | A>G | Yes | ATP6 | Y221C | Probably damaging | Primary PCA | M44 | Whole mtDNA | McCrow et al. 2015 |
| 9208 | T>C | Yes | COX3 | M1T | Possibly damaging | Primary PCA | LIND-SWE-32 | Whole mtDNA | Lindberg et al. 2013 |
| 9233 | T>C | Yes | COX3 | H9H | Synonymous SNV | Primary PCA | AK-5980 | Whole mtDNA | Kalsbeek et al. 2016 |
| 9305 | G>A | Yes | COX3 | M33M | Synonymous SNV | Primary PCA | JU-150 | Whole mtDNA | Ju et al. 2014 |
| 9307 | G>A | Yes | COX3 | W34X | Stopgain | Primary PCA | JU-136 | Whole mtDNA | Ju et al. 2014 |
| 9343 | G>A | Yes | COX3 | G46D | Possibly damaging | Primary PCA | M14 | Whole mtDNA | McCrow et al. 2015 |
| 9377 | G>A | Yes | COX3 | W57W | Synonymous SNV | Primary PCA | AR-140 | Whole mtDNA | Arnold et al. 2015 |
| 9377 | G>A | No | COX3 | W57W | Synonymous SNV | Bone Metastasis | AR-140 | Whole mtDNA | Arnold et al. 2015 |
| 9405 | NA | NA | COX3 | NA | NA | Primary PCA | NA | Whole mtDNA | Parr et al. 2006 |
| 9411 | G>A | Yes | COX3 | G69S | Probably damaging | Primary PCA | JU-44 | Whole mtDNA | Ju et al. 2014 |
| 9412 | G>A | Yes | COX3 | G69D | Probably damaging | Primary PCA | M23 | Whole mtDNA | McCrow et al. 2015 |
| 9438 | G>A | Yes | COX3 | G78S | Benign | Primary PCA | KB-PCA018 | Whole mtDNA | Kloss-Brandstatter et al. 2010 |
| 9474 | G>A | Yes | COX3 | E90K | Probably damaging | Primary PCA | LIND-SWE-13 | Whole mtDNA | Lindberg et al. 2013 |
| 9477 | G>C | Yes | COX3 | V91L | Benign | Soft Tissue Metastasis | AR-069 | Whole mtDNA | Arnold et al. 2015 |
| 9477 | NA | NA | COX3 | NA | NA | Primary PCA | NA | Whole mtDNA | Parr et al. 2006 |
| 9478 | T>A | Yes | COX3 | V91D | Possibly damaging | Primary PCA | AK-6080 | Whole mtDNA | Kalsbeek et al. 2016 |
| 9502 | NA | NA | COX3 | NA | NA | Primary PCA | NA | Whole mtDNA | Parr et al. 2006 |
| 9531 | A>AC | Yes | COX3 | Frameshift | Frameshift | Primary PCA | JU-129 | Whole mtDNA | Ju et al. 2014 |
| 9548 | G>A | Yes | COX3 | G114G | Synonymous SNV | Primary PCA | LIND-SWE-25 | Whole mtDNA | Lindberg et al. 2013 |
| 9564 | NA | NA | COX3 | NA | NA | Primary PCA | NA | Whole mtDNA | Parr et al. 2006 |
| 9565 | G>A | Yes | COX3 | G120D | Probably damaging | Primary PCA | JU-131 | Whole mtDNA | Ju et al. 2014 |
| 9574 | NA | NA | COX3 | NA | NA | Primary PCA | NA | Whole mtDNA | Parr et al. 2006 |
| 9591 | G>A | Yes | COX3 | V129I | Possibly damaging | Bone Metastasis | AR-050 | Whole mtDNA | Arnold et al. 2015 |
| 9595 | C>T | Yes | COX3 | P130L | Probably damaging | Primary PCA | JU-1286 | Whole mtDNA | Ju et al. 2014 |
| 9627 | G>A | Yes | COX3 | G141X | Stopgain | Primary PCA | JU-1157 | Whole mtDNA | Ju et al. 2014 |
| 9628 | NA | NA | COX3 | NA | NA | Primary PCA | NA | Whole mtDNA | Parr et al. 2006 |
| 9655 | G>A | Yes | COX3 | S150N | Possibly damaging | Primary PCA | JU-143 | Whole mtDNA | Ju et al. 2014 |
| 9673 | G>A | Yes | COX3 | R156Q | Probably damaging | Primary PCA | LIND-SWE-10 | Whole mtDNA | Lindberg et al. 2013 |
| 9820 | G>A | Yes | COX3 | G205E | Probably damaging | Primary PCA | AR-148 | Whole mtDNA | Arnold et al. 2015 |
| 9820 | G>A | Yes | COX3 | G205E | Probably damaging | Soft Tissue Metastasis | AR-148 | Whole mtDNA | Arnold et al. 2015 |
| 9820 | G>A | No | COX3 | G205E | Probably damaging | Bone Metastasis | AR-148 | Whole mtDNA | Arnold et al. 2015 |
| 9830 | C>T | Yes | COX3 | V208V | Synonymous SNV | Primary PCA | M23 | Whole mtDNA | McCrow et al. 2015 |
| 9893 | C>T | Yes | COX3 | S229S | Synonymous SNV | Primary PCA | M28 | Whole mtDNA | McCrow et al. 2015 |
| 9899 | T>C | Yes | COX3 | H231H | Synonymous SNV | Bone Metastasis | AR-050 | Whole mtDNA | Arnold et al. 2015 |
| 9899 | T>C | Yes | COX3 | H231H | Synonymous SNV | Soft Tissue Metastasis | AR-069 | Whole mtDNA | Arnold et al. 2015 |
| 9899 | C>T | Yes | COX3 | H231H | Synonymous SNV | Soft Tissue Metastasis | AR-108 | Whole mtDNA | Arnold et al. 2015 |
| 9899 | T>C | No | COX3 | H231H | Synonymous SNV | Bone Metastasis | AR-380 | Whole mtDNA | Arnold et al. 2015 |
| 9915 | G>A | Yes | COX3 | A237T | Probably damaging | Primary PCA | LIND-SWE-6 | Whole mtDNA | Lindberg et al. 2013 |
| 9925 | G>A | Yes | COX3 | W240X | Stopgain | Primary PCA | JU-183 | Whole mtDNA | Ju et al. 2014 |
| 9930 | T>C | Yes | COX3 | W242R | Probably damaging | Primary PCA | KB-PCA025 | Whole mtDNA | Kloss-Brandstatter et al. 2010 |
| 9938 | T>C | Yes | COX3 | F244F | Synonymous SNV | Primary PCA | JU-194 | Whole mtDNA | Ju et al. 2014 |
| 9949 | T>C | Yes | COX3 | V248A | Possibly damaging | Primary PCA | M16 | Whole mtDNA | McCrow et al. 2015 |
| 9962 | G>A | Yes | COX3 | L252L | Synonymous SNV | Primary PCA | AK-11129 | Whole mtDNA | Kalsbeek et al. 2016 |
| 9984 | G>A | Yes | COX3 | G260X | Stopgain | Primary PCA | JU-1362 | Whole mtDNA | Ju et al. 2014 |
| 10115 | T>C | Yes | ND3 | I19I | Synonymous SNV | Primary PCA | KB-PCA009 | Whole mtDNA | Kloss-Brandstatter et al. 2010 |
| 10138 | T>C | Yes | ND3 | L27P | Probably damaging | Primary PCA | JU-197 | Whole mtDNA | Ju et al. 2014 |
| 10143 | NA | NA | ND3 | NA | Noncoding | Primary PCA | NA | Whole mtDNA | Parr et al. 2006 |
| 10170 | G>C | Yes | ND3 | E38Q | Probably damaging | Bone Metastasis | SM001 | Whole mtDNA | Hovens et al. 2017 |
| 10203 | G>A | Yes | ND3 | V49I | benign | Bone Metastasis | AR-149 | Whole mtDNA | Arnold et al. 2015 |
| 10211 | T>C | No | ND3 | F51F | Synonymous SNV | Bone Metastasis | AR-148 | Whole mtDNA | Arnold et al. 2015 |
| 10238 | T>C | No | ND3 | I60I | Noncoding | Bone Metastasis | AR-149 | Whole mtDNA | Arnold et al. 2015 |
| 10260 | G>A | Yes | ND3 | E68K | Possibly damaging | Primary PCA | LIND-SWE-3 | Whole mtDNA | Lindberg et al. 2013 |
| 10290 | G>A | Yes | ND3 | A78T | Probably damaging | Primary PCA | LIND-SWE-15 | Whole mtDNA | Lindberg et al. 2013 |
| 10318 | T>C | Yes | ND3 | M87T | Benign | Primary PCA | JU-1363 | Whole mtDNA | Ju et al. 2014 |
| 10361 | T>C | Yes | ND3 | S101S | Synonymous SNV | Primary PCA | AK-5230 | Whole mtDNA | Kalsbeek et al. 2016 |
| 10378 | T>C | Yes | ND3 | L107P | Benign | Primary PCA | AK-6045 | Whole mtDNA | Kalsbeek et al. 2016 |
| 10387 | G>A | Yes | ND3 | G110E | Probably damaging | Primary PCA | JU-134 | Whole mtDNA | Ju et al. 2014 |
| 10398 | A>G | Yes | ND3 | T114A | benign | Bone Metastasis | AR-140 | Whole mtDNA | Arnold et al. 2015 |
| 10398 | A>G | Yes | ND3 | T114A | benign | Bone Metastasis | AR-165 | Whole mtDNA | Arnold et al. 2015 |
| 10398 | A>G | Yes | ND3 | T114A | benign | Bone Metastasis | AR-214 | Whole mtDNA | Arnold et al. 2015 |
| 10398 | A>G | Yes | ND3 | T114A | benign | Bone Metastasis | AR-380 | Whole mtDNA | Arnold et al. 2015 |
| 10398 | A>G | No | ND3 | T114A | benign | Bone Metastasis | AR-050 | Whole mtDNA | Arnold et al. 2015 |
| 10398 | A>G | No | ND3 | T114A | benign | Bone Metastasis | AR-148 | Whole mtDNA | Arnold et al. 2015 |
| 10398 | A>G | No | ND3 | T114A | benign | Bone Metastasis | AR-149 | Whole mtDNA | Arnold et al. 2015 |
| 10400 | C>T | No | ND3 | T114T | Synonymous SNV | Bone Metastasis | AR-380 | Whole mtDNA | Arnold et al. 2015 |
| 10436 | T>C | Yes | tRNA-Arg | Noncoding | tRNA | Primary PCA | KB-PCA017 | Whole mtDNA | Kloss-Brandstatter et al. 2010 |
| 10439 | NA | NA | tRNA-Arg | Noncoding | tRNA | Primary PCA | NA | Whole mtDNA | Parr et al. 2006 |
| 10455 | NA | NA | tRNA-Arg | Noncoding | tRNA | Primary PCA | NA | Whole mtDNA | Parr et al. 2006 |
| 10463 | C>T | Yes | tRNA-Arg | Noncoding | Noncoding | Bone Metastasis | AR-050 | Whole mtDNA | Arnold et al. 2015 |
| 10463 | T>C | No | tRNA-Arg | Noncoding | Noncoding | Bone Metastasis | AR-214 | Whole mtDNA | Arnold et al. 2015 |
| 10463 | T>C | No | tRNA-Arg | Noncoding | Noncoding | Bone Metastasis | AR-380 | Whole mtDNA | Arnold et al. 2015 |
| 10463 | C>T | No | tRNA-Arg | Noncoding | Noncoding | Soft Tissue Metastasis | AR-108 | Whole mtDNA | Arnold et al. 2015 |
| 10573 | G>A | Yes | ND4L | G35E | Probably damaging | Primary PCA | LIND-SWE-9 | Whole mtDNA | Lindberg et al. 2013 |
| 10587 | C>T | Yes | ND4L | L40L | Synonymous SNV | Primary PCA | JU-147 | Whole mtDNA | Ju et al. 2014 |
| 10588 | T>C | Yes | ND4L | L40P | Probably damaging | Primary PCA | JU-195 | Whole mtDNA | Ju et al. 2014 |
| 10589 | G>A | Yes | ND4L | L40L | Synonymous SNV | Bone Metastasis | AR-050 | Whole mtDNA | Arnold et al. 2015 |
| 10662 | G>A | Yes | ND4L | V65I | Benign | Primary PCA | JU-45 | Whole mtDNA | Ju et al. 2014 |
| 10688 | G>A | Yes | ND4L | V73V | Synonymous SNV | Primary PCA | AR-214 | Whole mtDNA | Arnold et al. 2015 |
| 10688 | G>A | Yes | ND4L | V73V | Synonymous SNV | Soft Tissue Metastasis | AR-214 | Whole mtDNA | Arnold et al. 2015 |
| 10695 | G>C | Yes | ND4L | A76P | Probably damaging | Primary PCA | LIND-SWE-37 | Whole mtDNA | Lindberg et al. 2013 |
| 10750 | G>A | No | ND4L | N94S | Benign | Bone Metastasis | AR-214 | Whole mtDNA | Arnold et al. 2015 |
| 10756 | T>C | Yes | ND4L | L96P | Probably damaging | Primary PCA | M32 | Whole mtDNA | McCrow et al. 2015 |
| 10756 | T>C | Yes | ND4L | L96P | Probably damaging | Primary PCA | M47 | Whole mtDNA | McCrow et al. 2015 |
| 10813 | CA>C | Yes | ND4 | Frameshift | Frameshift | Primary PCA | JU-134 | Whole mtDNA | Ju et al. 2014 |
| 10869 | T>C | Yes | ND4 | T521C | Probably damaging | Primary PCA | IC-DO36322 | Whole mtDNA | ICGC et al. 2016 |
| 10880 | T>C | Yes | ND4 | F41L | Probably damaging | Primary PCA | JU-180 | Whole mtDNA | Ju et al. 2014 |
| 10934 | G>A | Yes | ND4 | D59N | Probably damaging | Primary PCA | JU-1155 | Whole mtDNA | Ju et al. 2014 |
| 10987 | A>T | Yes | ND4 | T76T | Synonymous SNV | Primary PCA | JU-181 | Whole mtDNA | Ju et al. 2014 |
| 11010 | T>C | Yes | ND4 | L84S | Probably damaging | Primary PCA | M34 | Whole mtDNA | McCrow et al. 2015 |
| 11026 | A>G | Yes | ND4 | L89L | Synonymous SNV | Primary PCA | LIND-SWE-42 | Whole mtDNA | Lindberg et al. 2013 |
| 11032 | 7A>6A | No | ND4 | Frameshift | frameshift | Primary PCA | JE-46 | Targeted Seq | Jeronimo et al. 2001 |
| 11069 | NA | NA | ND4 | NA | Noncoding | Primary PCA | NA | Whole mtDNA | Parr et al. 2006 |
| 11120 | T>C | Yes | ND4 | F121L | benign | Soft Tissue Metastasis | AR-006 | Whole mtDNA | Arnold et al. 2015 |
| 11139 | T>C | Yes | ND4 | I127T | Probably damaging | Primary PCA | KB-PCA008 | Whole mtDNA | Kloss-Brandstatter et al. 2010 |
| 11166 | G>A | Yes | ND4 | W136X | Stopgain | Primary PCA | JU-825 | Whole mtDNA | Ju et al. 2014 |
| 11177 | NA | NA | ND4 | NA | Noncoding | Primary PCA | NA | Whole mtDNA | Parr et al. 2006 |
| 11197 | C>T | No | ND4 | G146G | Synonymous SNV | Bone Metastasis | AR-006 | Whole mtDNA | Arnold et al. 2015 |
| 11217 | NA | NA | ND4 | NA | Noncoding | Primary PCA | NA | Whole mtDNA | Parr et al. 2006 |
| 11225 | G>A | Yes | ND4 | G156S | Probably damaging | Primary PCA | M37 | Whole mtDNA | McCrow et al. 2015 |
| 11246 | G>A | Yes | ND4 | A163T | Probably damaging | Primary PCA | JU-1363 | Whole mtDNA | Ju et al. 2014 |
| 11246 | G>A | Yes | ND4 | A163T | Probably damaging | Primary PCA | LIND-SWE-3 | Whole mtDNA | Lindberg et al. 2013 |
| 11251 | A>G | Yes | ND4 | L164L | Synonymous SNV | Bone Metastasis | AR-050 | Whole mtDNA | Arnold et al. 2015 |
| 11251 | A>G | Yes | ND4 | L164L | Synonymous SNV | Soft Tissue Metastasis | AR-069 | Whole mtDNA | Arnold et al. 2015 |
| 11251 | A>G | No | ND4 | L164L | Synonymous SNV | Soft Tissue Metastasis | AR-108 | Whole mtDNA | Arnold et al. 2015 |
| 11280 | T>C | Yes | ND4 | L174L | Probably damaging | Primary PCA | IC-DO36269 | Whole mtDNA | ICGC et al. 2016 |
| 11351 | G>A | Yes | ND4 | A198T | Probably damaging | Primary PCA | AR-148 | Whole mtDNA | Arnold et al. 2015 |
| 11351 | G>A | Yes | ND4 | A198T | Probably damaging | Primary PCA | M16 | Whole mtDNA | McCrow et al. 2015 |
| 11351 | G>A | No | ND4 | A198T | Probably damaging | Soft Tissue Metastasis | AR-148 | Whole mtDNA | Arnold et al. 2015 |
| 11383 | T>C | Yes | ND4 | P208P | Synonymous SNV | Primary PCA | IC-DO36308 | Whole mtDNA | ICGC et al. 2016 |
| 11391 | G>A | Yes | ND4 | G211E | Probably damaging | Primary PCA | KB-PCA019 | Whole mtDNA | Kloss-Brandstatter et al. 2010 |
| 11410 | T>C | Yes | ND4 | P217P | Synonymous SNV | Primary PCA | AK-5288 | Whole mtDNA | Kalsbeek et al. 2016 |
| 11414 | G>A | Yes | ND4 | A219T | Probably damaging | Primary PCA | JU-1284 | Whole mtDNA | Ju et al. 2014 |
| 11420 | NA | NA | ND4 | NA | Noncoding | Primary PCA | NA | Whole mtDNA | Parr et al. 2006 |
| 11423 | G>A | Yes | ND4 | E222K | Probably damaging | Primary PCA | M24 | Whole mtDNA | McCrow et al. 2015 |
| 11435 | G>A | No | ND4 | A226T | Probably damaging | Primary PCA | AR-050 | Whole mtDNA | Arnold et al. 2015 |
| 11453 | G>A | Yes | ND4 | A232T | Probably damaging | Primary PCA | M55 | Whole mtDNA | McCrow et al. 2015 |
| 11467 | A>G | Yes | ND4 | A237A | Synonymous SNV | Primary PCA | LIND-SWE-24 | Whole mtDNA | Lindberg et al. 2013 |
| 11493 | G>A | Yes | ND4 | R245H | Probably damaging | Primary PCA | JU-152 | Whole mtDNA | Ju et al. 2014 |
| 11674 | C>T | No | ND4 | T305T | Synonymous SNV | Primary PCA | JE-01 | Targeted Seq | Jeronimo et al. 2001 |
| 11711 | G>A | Yes | ND4 | A318T | Probably damaging | Primary PCA | JU-200 | Whole mtDNA | Ju et al. 2014 |
| 11719 | G>A | Yes | ND4 | G320G | Synonymous SNV | Bone Metastasis | AR-050 | Whole mtDNA | Arnold et al. 2015 |
| 11719 | G>A | Yes | ND4 | G320G | Synonymous SNV | Primary PCA | AR-140 | Whole mtDNA | Arnold et al. 2015 |
| 11719 | G>A | Yes | ND4 | G320G | Synonymous SNV | Primary PCA | LIND-SWE-37 | Whole mtDNA | Lindberg et al. 2013 |
| 11719 | G>A | Yes | ND4 | G320G | Synonymous SNV | Soft Tissue Metastasis | AR-069 | Whole mtDNA | Arnold et al. 2015 |
| 11719 | G>A | No | ND4 | G320G | Synonymous SNV | Soft Tissue Metastasis | AR-108 | Whole mtDNA | Arnold et al. 2015 |
| 11770 | T>C | Yes | ND4 | T337T | Synonymous SNV | Primary PCA | M26 | Whole mtDNA | McCrow et al. 2015 |
| 11775 | G>A | Yes | ND4 | T1232C | Probably damaging | Primary PCA | IC-DO36264 | Whole mtDNA | ICGC et al. 2016 |
| 11775 | G>A | Yes | ND4 | S339N | Probably damaging | Primary PCA | JU-1283 | Whole mtDNA | Ju et al. 2014 |
| 11787 | T>C | Yes | ND4 | I343T | Probably damaging | Primary PCA | M43 | Whole mtDNA | McCrow et al. 2015 |
| 11799 | G>A | Yes | ND4 | G347E | Probably damaging | Primary PCA | JU-131 | Whole mtDNA | Ju et al. 2014 |
| 11812 | G>A | No | ND4 | L351L | Synonymous SNV | Bone Metastasis | AR-214 | Whole mtDNA | Arnold et al. 2015 |
| 11852 | NA | NA | ND4 | NA | Noncoding | Primary PCA | NA | Whole mtDNA | Parr et al. 2006 |
| 11889 | G>A | Yes | ND4 | G377E | Probably damaging | Primary PCA | JU-154 | Whole mtDNA | Ju et al. 2014 |
| 11907 | T>C | Yes | ND4 | V383A | Benign | Primary PCA | JU-130 | Whole mtDNA | Ju et al. 2014 |
| 11907 | NA | NA | ND4 | NA | Noncoding | Primary PCA | NA | Whole mtDNA | Parr et al. 2006 |
| 11921 | T>C | Yes | ND4 | W388R | Probably damaging | Primary PCA | KB-PCA001 | Whole mtDNA | Kloss-Brandstatter et al. 2010 |
| 11937 | T>C | Yes | ND4 | L393P | Possibly damaging | Primary PCA | JU-131 | Whole mtDNA | Ju et al. 2014 |
| 11947 | A>G | No | ND4 | T396T | Synonymous SNV | Primary PCA | JE-01 | Targeted Seq | Jeronimo et al. 2001 |
| 11956 | C>T | Yes | ND4 | N399N | Synonymous SNV | Primary PCA | JU-1364 | Whole mtDNA | Ju et al. 2014 |
| 11991 | T>C | Yes | ND4 | G473A | Benign | Primary PCA | IC-DO36351 | Whole mtDNA | ICGC et al. 2016 |
| 12009 | G>A | Yes | ND4 | G417D | Probably damaging | BPH | Pedi | Whole mtDNA | McCrow et al. 2015 |
| 12012 | NA | NA | ND4 | NA | Noncoding | Primary PCA | NA | Whole mtDNA | Parr et al. 2006 |
| 12186 | G>A | Yes | tRNA-His | Noncoding | tRNA | Primary PCA | JU-188 | Whole mtDNA | Ju et al. 2014 |
| 12280 | A>G | Yes | tRNA-Leu2 | Noncoding | tRNA | Primary PCA | AK-5288 | Whole mtDNA | Kalsbeek et al. 2016 |
| 12295 | T>C | Yes | tRNA-Leu2 | Noncoding | tRNA | Primary PCA | JU-127 | Whole mtDNA | Ju et al. 2014 |
| 12308 | A>G | No | tRNA-Leu2 | Noncoding | Noncoding | Primary PCA | AR-165 | Whole mtDNA | Arnold et al. 2015 |
| 12308 | A>G | No | tRNA-Leu2 | Noncoding | Noncoding | Primary PCA | JE-01 | Targeted Seq | Jeronimo et al. 2001 |
| 12308 | A>G | No | tRNA-Leu2 | Noncoding | Noncoding | Soft Tissue Metastasis | AR-165 | Whole mtDNA | Arnold et al. 2015 |
| 12316 | G>A | Yes | tRNA-Leu2 | Noncoding | tRNA | Primary PCA | KB-PCA001 | Whole mtDNA | Kloss-Brandstatter et al. 2010 |
| 12372 | G>A | Yes | ND5 | L12L | Synonymous SNV | Primary PCA | LIND-SWE-4C | Whole mtDNA | Lindberg et al. 2013 |
| 12372 | G>A | Yes | ND5 | L12L | Noncoding | Soft Tissue Metastasis | AR-165 | Whole mtDNA | Arnold et al. 2015 |
| 12372 | G>A | No | ND5 | L12L | Synonymous SNV | Primary PCA | AR-165 | Whole mtDNA | Arnold et al. 2015 |
| 12372 | G>A | No | ND5 | L12L | Synonymous SNV | Primary PCA | JE-01 | Targeted Seq | Jeronimo et al. 2001 |
| 12414 | T>C | No | ND5 | P26P | Synonymous SNV | Primary PCA | JE-01 | Targeted Seq | Jeronimo et al. 2001 |
| 12417 | CA>C | Yes | ND5 | Frameshift | Frameshift | Primary PCA | JU-130 | Whole mtDNA | Ju et al. 2014 |
| 12417 | C>CA | Yes | ND5 | Frameshift | Frameshift | Primary PCA | JU-45 | Whole mtDNA | Ju et al. 2014 |
| 12457 | G>A | Yes | ND5 | A41T | Probably damaging | Primary PCA | AK-11114 | Whole mtDNA | Kalsbeek et al. 2016 |
| 12457 | G>A | Yes | ND5 | A41T | Probably damaging | Primary PCA | M52 | Whole mtDNA | McCrow et al. 2015 |
| 12477 | T>C | Yes | ND5 | S47S | Synonymous SNV | Primary PCA | LIND-SWE-28 | Whole mtDNA | Lindberg et al. 2013 |
| 12501 | A>G | No | ND5 | M55M | Synonymous SNV | Bone Metastasis | AR-149 | Whole mtDNA | Arnold et al. 2015 |
| 12517 | G>A | Yes | ND5 | V61I | Benign | Primary PCA | JU-1285 | Whole mtDNA | Ju et al. 2014 |
| 12561 | G>A | Yes | ND5 | S77S | Synonymous SNV | Primary PCA | LIND-SWE-13 | Whole mtDNA | Lindberg et al. 2013 |
| 12601 | T>C | Yes | ND5 | F89L | Probably damaging | Primary PCA | LIND-SWE-20 | Whole mtDNA | Lindberg et al. 2013 |
| 12633 | C>A | Yes | ND5 | S99S | Synonymous SNV | Bone Metastasis | AR-050 | Whole mtDNA | Arnold et al. 2015 |
| 12705 | C>T | No | ND5 | I123I | Synonymous SNV | Primary PCA | JE-01 | Targeted Seq | Jeronimo et al. 2001 |
| 12730 | G>A | Yes | ND5 | V132I | Benign | Primary PCA | AK-11830 | Whole mtDNA | Kalsbeek et al. 2016 |
| 12757 | T>C | Yes | ND5 | F141L | Probably damaging | Primary PCA | JU-173 | Whole mtDNA | Ju et al. 2014 |
| 12758 | T>C | Yes | ND5 | F141S | Probably damaging | Primary PCA | M19 | Whole mtDNA | McCrow et al. 2015 |
| 12773 | G>A | Yes | ND5 | T621C | Probably damaging | Primary PCA | IC-DO36294 | Whole mtDNA | ICGC et al. 2016 |
| 12793 | T>C | Yes | ND5 | L153L | Synonymous SNV | BPH | Pedi | Whole mtDNA | McCrow et al. 2015 |
| 12818 | G>A | Yes | ND5 | R161Q | Probably damaging | BPH | Coloured | Whole mtDNA | McCrow et al. 2015 |
| 12853 | C>T | Yes | ND5 | L173L | Synonymous SNV | Primary PCA | M56 | Whole mtDNA | McCrow et al. 2015 |
| 12864 | T>C | Yes | ND5 | R176R | Synonymous SNV | Primary PCA | M43 | Whole mtDNA | McCrow et al. 2015 |
| 12871 | G>A | Yes | ND5 | D179N | Probably damaging | Primary PCA | M20 | Whole mtDNA | McCrow et al. 2015 |
| 12957 | T>C | Yes | ND5 | N207N | Synonymous SNV | Primary PCA | IC-DO36380 | Whole mtDNA | ICGC et al. 2016 |
| 12959 | NA | NA | ND5 | NA | Noncoding | Primary PCA | NA | Whole mtDNA | Parr et al. 2006 |
| 12994 | G>A | No | ND5 | A220T | Probably damaging | Primary PCA | AR-214 | Whole mtDNA | Arnold et al. 2015 |
| 12994 | G>A | No | ND5 | A220T | Probably damaging | Soft Tissue Metastasis | AR-214 | Whole mtDNA | Arnold et al. 2015 |
| 13019 | G>A | Yes | ND5 | G228D | Probably damaging | Bone Metastasis | SM002 | Whole mtDNA | Hovens et al. 2017 |
| 13105 | NA | NA | ND5 | NA | Noncoding | Primary PCA | NA | Whole mtDNA | Parr et al. 2006 |
| 13118 | T>G | NA | ND5 | I261S | Probably damaging | Primary PCA | IC-DO36301 | Whole mtDNA | ICGC et al. 2016 |
| 13198 | G>A | Yes | ND5 | A288T | Benign | Bone Metastasis | SM498 | Whole mtDNA | Hovens et al. 2017 |
| 13201 | G>A | Yes | ND5 | A289T | Probably damaging | Primary PCA | AK-5336 | Whole mtDNA | Kalsbeek et al. 2016 |
| 13203 | G>A | No | ND5 | A289E | Synonymous SNV | Bone Metastasis | AR-140 | Whole mtDNA | Arnold et al. 2015 |
| 13212 | C>T | Yes | ND5 | A292A | Synonymous SNV | Primary PCA | M39 | Whole mtDNA | McCrow et al. 2015 |
| 13294 | NA | NA | ND5 | NA | Noncoding | Primary PCA | NA | Whole mtDNA | Parr et al. 2006 |
| 13322 | T>C | Yes | ND5 | I329T | Probably damaging | Primary PCA | JU-196 | Whole mtDNA | Ju et al. 2014 |
| 13326 | T>C | Yes | ND5 | C330C | Synonymous SNV | Primary PCA | IC-DO36296 | Whole mtDNA | ICGC et al. 2016 |
| 13367 | G>A | Yes | ND5 | G344E | Probably damaging | Primary PCA | JU-193 | Whole mtDNA | Ju et al. 2014 |
| 13367 | G>A | NA | ND5 | G344E | Probably damaging | Primary PCA | IC-DO36362 | Whole mtDNA | ICGC et al. 2016 |
| 13368 | G>A | Yes | ND5 | G344G | Synonymous SNV | Primary PCA | LIND-SWE-34 | Whole mtDNA | Lindberg et al. 2013 |
| 13368 | A>G | No | ND5 | G344G | Synonymous SNV | Bone Metastasis | AR-380 | Whole mtDNA | Arnold et al. 2015 |
| 13416 | A>G | Yes | ND5 | G360G | Synonymous SNV | Primary PCA | AK-5874 | Whole mtDNA | Kalsbeek et al. 2016 |
| 13463 | G>A | Yes | ND5 | G376D | Probably damaging | Primary PCA | JU-141 | Whole mtDNA | Ju et al. 2014 |
| 13464 | C>T | Yes | ND5 | G376G | Synonymous SNV | Primary PCA | IC-DO36271 | Whole mtDNA | ICGC et al. 2016 |
| 13481 | G>A | Yes | ND5 | G382E | Probably damaging | Primary PCA | M43 | Whole mtDNA | McCrow et al. 2015 |
| 13484 | NA | NA | ND5 | NA | Noncoding | Primary PCA | NA | Whole mtDNA | Parr et al. 2006 |
| 13488 | T>G | Yes | ND5 | P384P | Synonymous SNV | Primary PCA | KB-PCA002 | Whole mtDNA | Kloss-Brandstatter et al. 2010 |
| 13513 | G>A | Yes | ND5 | D393N | Probably damaging | Bone Metastasis | SM067 | Whole mtDNA | Hovens et al. 2017 |
| 13531 | G>A | Yes | ND5 | A399T | Probably damaging | BPH | Pedi | Whole mtDNA | McCrow et al. 2015 |
| 13552 | G>A | Yes | ND5 | A406T | Benign | Primary PCA | JU-185 | Whole mtDNA | Ju et al. 2014 |
| 13617 | T>C | Yes | ND5 | I427I | Synonymous SNV | Soft Tissue Metastasis | AR-069 | Whole mtDNA | Arnold et al. 2015 |
| 13634 | NA | NA | ND5 | NA | Noncoding | Primary PCA | NA | Whole mtDNA | Parr et al. 2006 |
| 13680 | C>T | Yes | ATP6\|ATP8 | P448P | Synonymous SNV | Primary PCA | IC-DO36296 | Whole mtDNA | ICGC et al. 2016 |
| 13685 | T>C | Yes | ND5 | L450P | Probably damaging | Primary PCA | M26 | Whole mtDNA | McCrow et al. 2015 |
| 13703 | G>A | Yes | ND5 | R456H | Probably damaging | Primary PCA | AK-5066 | Whole mtDNA | Kalsbeek et al. 2016 |
| 13711 | G>A | Yes | ND5 | A459T | Benign | Primary PCA | IC-DO36252 | Whole mtDNA | ICGC et al. 2016 |
| 13718 | G>A | Yes | ND5 | S461N | Probably damaging | Primary PCA | KB-PCA023 | Whole mtDNA | Kloss-Brandstatter et al. 2010 |
| 13723 | T>C | Yes | ND5 | F463L | benign | Primary PCA | AR-148 | Whole mtDNA | Arnold et al. 2015 |
| 13723 | T>C | No | ND5 | F463L | benign | Bone Metastasis | AR-148 | Whole mtDNA | Arnold et al. 2015 |
| 13723 | T>C | No | ND5 | F463L | benign | Soft Tissue Metastasis | AR-148 | Whole mtDNA | Arnold et al. 2015 |
| 13758 | C>A | Yes | ND5 | P474P | Synonymous SNV | Primary PCA | M15 | Whole mtDNA | McCrow et al. 2015 |
| 13759 | NA | NA | ND5 | NA | Noncoding | Primary PCA | NA | Whole mtDNA | Parr et al. 2006 |
| 13763 | C>G | Yes | ND5 | S476C | Probably damaging | Primary PCA | AK-6108 | Whole mtDNA | Kalsbeek et al. 2016 |
| 13789 | NA | NA | ND5 | NA | Noncoding | Primary PCA | NA | Whole mtDNA | Parr et al. 2006 |
| 13805 | NA | NA | ND5 | NA | Noncoding | Primary PCA | NA | Whole mtDNA | Parr et al. 2006 |
| 13830 | T>C | Yes | ND5 | L498L | Synonymous SNV | Bone Metastasis | AR-165 | Whole mtDNA | Arnold et al. 2015 |
| 13832 | T>C | Yes | ND5 | L499P | Probably damaging | Primary PCA | LIND-SWE-15 | Whole mtDNA | Lindberg et al. 2013 |
| 13880 | NA | NA | ND5 | NA | Noncoding | Primary PCA | NA | Whole mtDNA | Parr et al. 2006 |
| 13933 | NA | NA | ND5 | NA | Noncoding | Primary PCA | NA | Whole mtDNA | Parr et al. 2006 |
| 13979 | T>C | Yes | ND5 | L548P | Probably damaging | Primary PCA | JU-143 | Whole mtDNA | Ju et al. 2014 |
| 13994 | T>C | Yes | ND5 | L553P | Probably damaging | Primary PCA | LIND-SWE-35 | Whole mtDNA | Lindberg et al. 2013 |
| 14044 | NA | NA | ND5 | NA | Noncoding | Primary PCA | NA | Whole mtDNA | Parr et al. 2006 |
| 14053 | A>G | No | ND5 | T573A | Benign | Primary PCA | JE-01 | Targeted Seq | Jeronimo et al. 2001 |
| 14097 | C>T | Yes | ND5 | Y587Y | Synonymous SNV | Primary PCA | IC-DO36368 | Whole mtDNA | ICGC et al. 2016 |
| 14132 | T>C | NA | ND5 | L599P | Possibly damaging | Primary PCA | IC-DO49810 | Whole mtDNA | ICGC et al. 2016 |
| 14178 | NA | NA | ND6 | NA | Noncoding | Primary PCA | NA | Whole mtDNA | Parr et al. 2006 |
| 14221 | T>C | Yes | ND6 | W151W | Synonymous SNV | Primary PCA | KB-PCA030 | Whole mtDNA | Kloss-Brandstatter et al. 2010 |
| 14233 | A>G | Yes | ND6 | D147D | Synonymous SNV | Bone Metastasis | AR-214 | Whole mtDNA | Arnold et al. 2015 |
| 14384 | G>A | Yes | ND6 | A97V | Benign | Primary PCA | LIND-SWE-16 | Whole mtDNA | Lindberg et al. 2013 |
| 14451 | T>C | Yes | ND6 | I75V | Benign | Primary PCA | M25 | Whole mtDNA | McCrow et al. 2015 |
| 14463 | T>C | Yes | ND6 | T71A | Benign | Primary PCA | KB-PCA025 | Whole mtDNA | Kloss-Brandstatter et al. 2010 |
| 14547 | T>C | Yes | ND6 | I43V | Benign | Primary PCA | LIND-SWE-15 | Whole mtDNA | Lindberg et al. 2013 |
| 14560 | G>A | Yes | ND6 | V38V | Synonymous SNV | Primary PCA | AK-16995 | Whole mtDNA | Kalsbeek et al. 2016 |
| 14560 | G>A | Yes | ND6 | V38V | Synonymous SNV | Primary PCA | M48 | Whole mtDNA | McCrow et al. 2015 |
| 14582 | A>G | Yes | ND6 | V31A | Benign | Primary PCA | LIND-SWE-43 | Whole mtDNA | Lindberg et al. 2013 |
| 14598 | T>C | Yes | ND6 | I26V | Probably damaging | Primary PCA | AK-12708 | Whole mtDNA | Kalsbeek et al. 2016 |
| 14601 | G>A | Yes | ND6 | P25S | Probably damaging | Primary PCA | AK-5400 | Whole mtDNA | Kalsbeek et al. 2016 |
| 14607 | G>A | Yes | ND6 | P23S | Probably damaging | Primary PCA | LIND-SWE-3 | Whole mtDNA | Lindberg et al. 2013 |
| 14681 | G>A | Yes | tRNA-Glu | Noncoding | tRNA | Primary PCA | M20 | Whole mtDNA | McCrow et al. 2015 |
| 14750 | G>A | No | CYTB | T2A | Benign | Soft Tissue Metastasis | AR-069 | Whole mtDNA | Arnold et al. 2015 |
| 14766 | C>T | Yes | CYTB | T7I | benign | Bone Metastasis | AR-050 | Whole mtDNA | Arnold et al. 2015 |
| 14766 | C>T | Yes | CYTB | T7I | Benign | Primary PCA | LIND-SWE-54A | Whole mtDNA | Lindberg et al. 2013 |
| 14766 | C>T | Yes | CYTB | T7I | benign | Soft Tissue Metastasis | AR-069 | Whole mtDNA | Arnold et al. 2015 |
| 14769 | A>G | NA | CYTB | N8S | Probably damaging | Primary PCA | PE-t18 | Targeted Seq | Petros et al. 2005 |
| 14793 | A>G | Yes | CYTB | H16R | benign | Soft Tissue Metastasis | AR-006 | Whole mtDNA | Arnold et al. 2015 |
| 14840 | A>G | Yes | CYTB | N32D | Probably damaging | Primary PCA | AK-13104 | Whole mtDNA | Kalsbeek et al. 2016 |
| 14846 | G>A | Yes | CYTB | G34S | Probably damaging | Primary PCA | LIND-SWE-54A | Whole mtDNA | Lindberg et al. 2013 |
| 14849 | T>C | Yes | CYTB | S35P | Probably damaging | Bone Metastasis | SM498 | Whole mtDNA | Hovens et al. 2017 |
| 14858 | G>A | Yes | CYTB | G38S | Probably damaging | BPH | Pedi | Whole mtDNA | McCrow et al. 2015 |
| 14872 | C>T | Yes | CYTB | I42I | Synonymous SNV | Primary PCA | IC-DO36296 | Whole mtDNA | ICGC et al. 2016 |
| 14889 | G>A | Yes | CYTB | G48E | Probably damaging | Primary PCA | LIND-SWE-1 | Whole mtDNA | Lindberg et al. 2013 |
| 14903 | NA | NA | CYTB | NA | NA | Primary PCA | NA | Whole mtDNA | Parr et al. 2006 |
| 14972 | G>A | Yes | CYTB | G76S | Probably damaging | Primary PCA | LIND-SWE-16 | Whole mtDNA | Lindberg et al. 2013 |
| 15001 | T>C | Yes | CYTB | N85N | Synonymous SNV | Primary PCA | M23 | Whole mtDNA | McCrow et al. 2015 |
| 15059 | G>A | Yes | CYTB | G105X | Stopgain | Primary PCA | AK-13179 | Whole mtDNA | Kalsbeek et al. 2016 |
| 15110 | G>A | Yes | CYTB | A122T | Benign | Primary PCA | LIND-SWE-37 | Whole mtDNA | Lindberg et al. 2013 |
| 15153 | G>A | Yes | CYTB | G136D | Probably damaging | Primary PCA | JU-159 | Whole mtDNA | Ju et al. 2014 |
| 15162 | NA | NA | CYTB | NA | NA | Primary PCA | NA | Whole mtDNA | Parr et al. 2006 |
| 15184 | T>C | Yes | CYTB | I146I | Synonymous SNV | Primary PCA | JU-127 | Whole mtDNA | Ju et al. 2014 |
| 15204 | T>C | Yes | CYTB | I153T | Benign | Primary PCA | JU-187 | Whole mtDNA | Ju et al. 2014 |
| 15218 | A>G | Yes | CYTB | T158A | Possibly damaging | Primary PCA | JU-136 | Whole mtDNA | Ju et al. 2014 |
| 15243 | A>G | Yes | CYTB | G166G | Synonymous SNV | Primary PCA | KB-PCA019 | Whole mtDNA | Kloss-Brandstatter et al. 2010 |
| 15270 | T>C | Yes | CYTB | L175P | Probably damaging | Primary PCA | AK-5463 | Whole mtDNA | Kalsbeek et al. 2016 |
| 15288 | T>C | Yes | CYTB | F181S | Benign | Primary PCA | M30 | Whole mtDNA | McCrow et al. 2015 |
| 15297 | T>C | Yes | CYTB | I184T | Possibly damaging | Primary PCA | AK-17392 | Whole mtDNA | Kalsbeek et al. 2016 |
| 15302 | NA | NA | CYTB | NA | NA | Primary PCA | NA | Whole mtDNA | Parr et al. 2006 |
| 15313 | T>C | Yes | CYTB | I189I | Synonymous SNV | Primary PCA | KB-PCA017 | Whole mtDNA | Kloss-Brandstatter et al. 2010 |
| 15323 | NA | NA | CYTB | NA | NA | Primary PCA | NA | Whole mtDNA | Parr et al. 2006 |
| 15324 | NA | NA | CYTB | NA | NA | Primary PCA | NA | Whole mtDNA | Parr et al. 2006 |
| 15330 | T>C | Yes | CYTB | L195P | Probably damaging | Primary PCA | AK-5060 | Whole mtDNA | Kalsbeek et al. 2016 |
| 15357 | G>A | Yes | CYTB | G204E | Probably damaging | Primary PCA | AK-11590 | Whole mtDNA | Kalsbeek et al. 2016 |
| 15375 | G>A | Yes | CYTB | G210E | Probably damaging | Primary PCA | JU-159 | Whole mtDNA | Ju et al. 2014 |
| 15375 | G>A | Yes | CYTB | G210E | Probably damaging | Primary PCA | M12 | Whole mtDNA | McCrow et al. 2015 |
| 15384 | NA | NA | CYTB | NA | NA | Primary PCA | NA | Whole mtDNA | Parr et al. 2006 |
| 15452 | A>C | No | CYTB | L236I | Benign | Bone Metastasis | AR-380 | Whole mtDNA | Arnold et al. 2015 |
| 15500 | G>A | Yes | CYTB | D252N | Probably damaging | Primary PCA | JU-182 | Whole mtDNA | Ju et al. 2014 |
| 15500 | G>A | Yes | CYTB | D252N | Probably damaging | Primary PCA | M46 | Whole mtDNA | McCrow et al. 2015 |
| 15527 | NA | NA | CYTB | NA | NA | Primary PCA | NA | Whole mtDNA | Parr et al. 2006 |
| 15553 | G>A | Yes | CYTB | K269K | Synonymous SNV | BPH | Pedi | Whole mtDNA | McCrow et al. 2015 |
| 15575 | G>A | Yes | CYTB | A277T | Probably damaging | Primary PCA | JU-177 | Whole mtDNA | Ju et al. 2014 |
| 15591 | G>A | Yes | CYTB | R282Q | Probably damaging | Primary PCA | AK-5252 | Whole mtDNA | Kalsbeek et al. 2016 |
| 15607 | A>G | Yes | CYTB | K287K | Synonymous SNV | Soft Tissue Metastasis | AR-069 | Whole mtDNA | Arnold et al. 2015 |
| 15607 | G>A | No | CYTB | K287K | Synonymous SNV | Bone Metastasis | AR-380 | Whole mtDNA | Arnold et al. 2015 |
| 15629 | C>T | No | CYTB | L295L | Synonymous SNV | Primary PCA | AK-5958 | Whole mtDNA | Kalsbeek et al. 2016 |
| 15670 | T>C | Yes | CYTB | H308H | Synonymous SNV | Primary PCA | AK-12708 | Whole mtDNA | Kalsbeek et al. 2016 |
| 15674 | T>C | Yes | CYTB | S310P | Benign | Primary PCA | LIND-SWE-17 | Whole mtDNA | Lindberg et al. 2013 |
| 15693 | T>C | Yes | CYTB | M316T | Benign | Primary PCA | LIND-SWE-37 | Whole mtDNA | Lindberg et al. 2013 |
| 15755 | T>C | Yes | CYTB | W337R | Probably damaging | Primary PCA | JU-183 | Whole mtDNA | Ju et al. 2014 |
| 15761 | G>A | Yes | CYTB | G339X | Stopgain | Primary PCA | M56 | Whole mtDNA | McCrow et al. 2015 |
| 15884 | G>A | Yes | CYTB | A380T | benign | Bone Metastasis | AR-050 | Whole mtDNA | Arnold et al. 2015 |
| 15904 | T>C | No | tRNA-Thr | Noncoding | Noncoding | Soft Tissue Metastasis | AR-108 | Whole mtDNA | Arnold et al. 2015 |
| 15928 | G>A | Yes | tRNA-Thr | Noncoding | Noncoding | Bone Metastasis | AR-050 | Whole mtDNA | Arnold et al. 2015 |
| 15928 | G>A | Yes | tRNA-Thr | Noncoding | Noncoding | Soft Tissue Metastasis | AR-069 | Whole mtDNA | Arnold et al. 2015 |
| 15928 | G>A | No | tRNA-Thr | Noncoding | Noncoding | Soft Tissue Metastasis | AR-108 | Whole mtDNA | Arnold et al. 2015 |
| 15946 | NA | NA | tRNA-Pro | Noncoding | tRNA | Primary PCA | NA | Whole mtDNA | Parr et al. 2006 |
| 15959 | G>A | Yes | tRNA-Pro | Noncoding | tRNA | Primary PCA | AK-5066 | Whole mtDNA | Kalsbeek et al. 2016 |
| 15995 | NA | NA | tRNA-Pro | Noncoding | tRNA | Primary PCA | NA | Whole mtDNA | Parr et al. 2006 |
| 15996 | NA | NA | tRNA-Pro | Noncoding | tRNA | Primary PCA | NA | Whole mtDNA | Parr et al. 2006 |
| 15998 | NA | NA | tRNA-Pro | Noncoding | tRNA | Primary PCA | NA | Whole mtDNA | Parr et al. 2006 |
| 15999 | NA | NA | tRNA-Pro | Noncoding | tRNA | Primary PCA | NA | Whole mtDNA | Parr et al. 2006 |
| 16000 | NA | NA | tRNA-Pro | Noncoding | tRNA | Primary PCA | NA | Whole mtDNA | Parr et al. 2006 |
| 16027 | T>C | Yes | D-Loop | Noncoding | Noncoding | Primary PCA | M37 | Whole mtDNA | McCrow et al. 2015 |
| 16034 | G>A | Yes | D-Loop | Noncoding | Noncoding | Primary PCA | JU-148 | Whole mtDNA | Ju et al. 2014 |
| 16034 | G>A | Yes | D-Loop | Noncoding | Noncoding | Primary PCA | JU-188 | Whole mtDNA | Ju et al. 2014 |
| 16035 | G>A | Yes | D-Loop | Noncoding | Noncoding | Primary PCA | AK-5684 | Whole mtDNA | Kalsbeek et al. 2016 |
| 16035 | G>A | Yes | D-Loop | Noncoding | Noncoding | Primary PCA | M17 | Whole mtDNA | McCrow et al. 2015 |
| 16047 | G>A | Yes | D-Loop | Noncoding | Noncoding | Primary PCA | KB-PCA005 | Whole mtDNA | Kloss-Brandstatter et al. 2010 |
| 16069 | C>A | No | D-Loop | Noncoding | Noncoding | Primary PCA | GZ-08 | Targeted Seq | Gomez-Zaera et al. 2006 |
| 16093 | T>C | Yes | D-Loop | Noncoding | Noncoding | Primary PCA | AK-12708 | Whole mtDNA | Kalsbeek et al. 2016 |
| 16093 | T>C | Yes | D-Loop | Noncoding | Noncoding | Primary PCA | AK-5064 | Whole mtDNA | Kalsbeek et al. 2016 |
| 16093 | C>T | No | D-Loop | Noncoding | Noncoding | Primary PCA | AK-11129 | Whole mtDNA | Kalsbeek et al. 2016 |
| 16093 | C>T | No | D-Loop | Noncoding | Noncoding | Primary PCA | CH-14 | Targeted Seq | Chen et al. 2002 |
| 16108 | C>T | Yes | D-Loop | Noncoding | Noncoding | Primary PCA | M30 | Whole mtDNA | McCrow et al. 2015 |
| 16111 | C>T | No | D-Loop | Noncoding | Noncoding | Primary PCA | CH-04 | Targeted Seq | Chen et al. 2002 |
| 16129 | A>G | No | D-Loop | Noncoding | Noncoding | Bone Metastasis | AR-149 | Whole mtDNA | Arnold et al. 2015 |
| 16147 | C>T | Yes | D-Loop | Noncoding | Noncoding | Primary PCA | JU-130 | Whole mtDNA | Ju et al. 2014 |
| 16148 | C>T | Yes | D-Loop | Noncoding | Noncoding | Bone Metastasis | SM001 | Whole mtDNA | Hovens et al. 2017 |
| 16148 | C>T | Yes | D-Loop | Noncoding | Noncoding | Primary PCA | M30 | Whole mtDNA | McCrow et al. 2015 |
| 16153 | G>A | Yes | D-Loop | Noncoding | Noncoding | Primary PCA | AK-15178 | Whole mtDNA | Kalsbeek et al. 2016 |
| 16172 | T>C | Yes | D-Loop | Noncoding | Noncoding | Soft Tissue Metastasis | AR-006 | Whole mtDNA | Arnold et al. 2015 |
| 16182 | A>C | Yes | D-Loop | Noncoding | Noncoding | Primary PCA | CH-01 | Targeted Seq | Chen et al. 2002 |
| 16182 | A>C | No | D-Loop | Noncoding | Noncoding | Primary PCA | CH-04 | Targeted Seq | Chen et al. 2002 |
| 16183 | A>AC | Yes | D-Loop | Noncoding | Noncoding | Bone Metastasis | SM067 | Whole mtDNA | Hovens et al. 2017 |
| 16183 | A>C | Yes | D-Loop | Noncoding | Noncoding | Primary PCA | CH-01 | Targeted Seq | Chen et al. 2002 |
| 16183 | A>AC | Yes | D-Loop | Noncoding | Noncoding | Primary PCA | JU-154 | Whole mtDNA | Ju et al. 2014 |
| 16183 | A>C | No | D-Loop | Noncoding | Noncoding | Primary PCA | CH-04 | Targeted Seq | Chen et al. 2002 |
| 16183 | A>G | No | D-Loop | Noncoding | Noncoding | Primary PCA | JE-01 | Targeted Seq | Jeronimo et al. 2001 |
| 16184 | 10C>9C | Yes | D-Loop | Noncoding | Noncoding | Primary PCA | CH-12 | Targeted Seq | Chen et al. 2002 |
| 16188 | CT>C | Yes | D-Loop | Noncoding | Noncoding | Primary PCA | JU-154 | Whole mtDNA | Ju et al. 2014 |
| 16189 | T>C | Yes | D-Loop | Noncoding | Noncoding | Primary PCA | CH-01 | Targeted Seq | Chen et al. 2002 |
| 16189 | T>C | Yes | D-Loop | Noncoding | Noncoding | Primary PCA | JU-154 | Whole mtDNA | Ju et al. 2014 |
| 16189 | T>C | No | D-Loop | Noncoding | Noncoding | Primary PCA | CH-04 | Targeted Seq | Chen et al. 2002 |
| 16189 | T>C | No | D-Loop | Noncoding | Noncoding | Primary PCA | JE-01 | Targeted Seq | Jeronimo et al. 2001 |
| 16192 | C>T | Yes | D-Loop | Noncoding | Noncoding | Soft Tissue Metastasis | AR-006 | Whole mtDNA | Arnold et al. 2015 |
| 16217 | T>C | No | D-Loop | Noncoding | Noncoding | Primary PCA | CH-04 | Targeted Seq | Chen et al. 2002 |
| 16224 | T>C | No | D-Loop | Noncoding | Noncoding | Primary PCA | AK-14919 | Whole mtDNA | Kalsbeek et al. 2016 |
| 16224 | C>T | No | D-Loop | Noncoding | Noncoding | Soft Tissue Metastasis | AR-069 | Whole mtDNA | Arnold et al. 2015 |
| 16232 | C>A | No | D-Loop | Noncoding | Noncoding | Primary PCA | CH-02 | Targeted Seq | Chen et al. 2002 |
| 16249 | T>C | Yes | D-Loop | Noncoding | Noncoding | Primary PCA | CH-01 | Targeted Seq | Chen et al. 2002 |
| 16250 | C>T | Yes | D-Loop | Noncoding | Noncoding | Primary PCA | AR-380 | Whole mtDNA | Arnold et al. 2015 |
| 16250 | C>T | Yes | D-Loop | Noncoding | Noncoding | Soft Tissue Metastasis | AR-380 | Whole mtDNA | Arnold et al. 2015 |
| 16256 | C>T | Yes | D-Loop | Noncoding | Noncoding | Soft Tissue Metastasis | AR-006 | Whole mtDNA | Arnold et al. 2015 |
| 16259 | C>T | Yes | D-Loop | Noncoding | Noncoding | Primary PCA | JU-175 | Whole mtDNA | Ju et al. 2014 |
| 16274 | G>A | Yes | D-Loop | Noncoding | Noncoding | Primary PCA | CH-01 | Targeted Seq | Chen et al. 2002 |
| 16278 | C>T | Yes | D-Loop | Noncoding | Noncoding | Primary PCA | JU-1283 | Whole mtDNA | Ju et al. 2014 |
| 16284 | A>G | Yes | D-Loop | Noncoding | Noncoding | Primary PCA | GZ-11 | Targeted Seq | Gomez-Zaera et al. 2006 |
| 16286 | C>T | Yes | D-Loop | Noncoding | Noncoding | Primary PCA | AK-5405 | Whole mtDNA | Kalsbeek et al. 2016 |
| 16290 | C>T | Yes | D-Loop | Noncoding | Noncoding | Primary PCA | AK-4148 | Whole mtDNA | Kalsbeek et al. 2016 |
| 16290 | C>T | Yes | D-Loop | Noncoding | Noncoding | Primary PCA | M11 | Whole mtDNA | McCrow et al. 2015 |
| 16293 | A>G | Yes | D-Loop | Noncoding | Noncoding | Primary PCA | AK-5132 | Whole mtDNA | Kalsbeek et al. 2016 |
| 16294 | C>T | Yes | D-Loop | Noncoding | Noncoding | Bone Metastasis | AR-050 | Whole mtDNA | Arnold et al. 2015 |
| 16294 | C>T | Yes | D-Loop | Noncoding | Noncoding | Primary PCA | M11 | Whole mtDNA | McCrow et al. 2015 |
| 16294 | C>T | Yes | D-Loop | Noncoding | Noncoding | Soft Tissue Metastasis | AR-069 | Whole mtDNA | Arnold et al. 2015 |
| 16296 | C>T | No | D-Loop | Noncoding | Noncoding | Primary PCA | AK-5405 | Whole mtDNA | Kalsbeek et al. 2016 |
| 16298 | T>C | Yes | D-Loop | Noncoding | Noncoding | Primary PCA | CH-01 | Targeted Seq | Chen et al. 2002 |
| 16298 | T>C | No | D-Loop | Noncoding | Noncoding | Primary PCA | CH-05 | Targeted Seq | Chen et al. 2002 |
| 16304 | T>C | Yes | D-Loop | Noncoding | Noncoding | Primary PCA | CH-01 | Targeted Seq | Chen et al. 2002 |
| 16311 | T>C | Yes | D-Loop | Noncoding | Noncoding | Primary PCA | CH-01 | Targeted Seq | Chen et al. 2002 |
| 16311 | C>T | No | D-Loop | Noncoding | Noncoding | Soft Tissue Metastasis | AR-069 | Whole mtDNA | Arnold et al. 2015 |
| 16320 | C>T | Yes | D-Loop | Noncoding | Noncoding | Primary PCA | JU-153 | Whole mtDNA | Ju et al. 2014 |
| 16320 | C>T | Yes | D-Loop | Noncoding | Noncoding | Soft Tissue Metastasis | AR-006 | Whole mtDNA | Arnold et al. 2015 |
| 16327 | C>T | Yes | D-Loop | Noncoding | Noncoding | Primary PCA | JU-144 | Whole mtDNA | Ju et al. 2014 |
| 16327 | C>T | Yes | D-Loop | Noncoding | Noncoding | Primary PCA | M30 | Whole mtDNA | McCrow et al. 2015 |
| 16355 | C>T | Yes | D-Loop | Noncoding | Noncoding | Primary PCA | M32 | Whole mtDNA | McCrow et al. 2015 |
| 16380 | C>T | Yes | D-Loop | Noncoding | Noncoding | Primary PCA | M11 | Whole mtDNA | McCrow et al. 2015 |
| 16380 | C>T | Yes | D-Loop | Noncoding | Noncoding | Primary PCA | M44 | Whole mtDNA | McCrow et al. 2015 |
| 16380 | C>T | Yes | D-Loop | Noncoding | Noncoding | Primary PCA | M56 | Whole mtDNA | McCrow et al. 2015 |
| 16390 | G>A | Yes | D-Loop | Noncoding | Noncoding | Primary PCA | M11 | Whole mtDNA | McCrow et al. 2015 |
| 16390 | G>A | Yes | D-Loop | Noncoding | Noncoding | Primary PCA | M30 | Whole mtDNA | McCrow et al. 2015 |
| 16399 | A>G | Yes | D-Loop | Noncoding | Noncoding | Primary PCA | M44 | Whole mtDNA | McCrow et al. 2015 |
| 16403 | C>T | No | D-Loop | Noncoding | Noncoding | Primary PCA | CH-11 | Targeted Seq | Chen et al. 2002 |
| 16459 | C>T | No | D-Loop | Noncoding | Noncoding | Primary PCA | CH-12 | Targeted Seq | Chen et al. 2002 |
| 16474 | G>C | Yes | D-Loop | Noncoding | Noncoding | Primary PCA | CH-13 | Targeted Seq | Chen et al. 2002 |
| 16519 | T>C | Yes | D-Loop | Noncoding | Noncoding | Bone Metastasis | AR-050 | Whole mtDNA | Arnold et al. 2015 |
| 16519 | T>C | Yes | D-Loop | Noncoding | Noncoding | Primary PCA | AK-5336 | Whole mtDNA | Kalsbeek et al. 2016 |
| 16519 | T>C | Yes | D-Loop | Noncoding | Noncoding | Primary PCA | JU-1283 | Whole mtDNA | Ju et al. 2014 |
| 16519 | T>C | No | D-Loop | Noncoding | Noncoding | Primary PCA | CH-05 | Targeted Seq | Chen et al. 2002 |
| 16519 | T>C | No | D-Loop | Noncoding | Noncoding | Soft Tissue Metastasis | AR-006 | Whole mtDNA | Arnold et al. 2015 |
| 16537 | C>T | Yes | D-Loop | Noncoding | Noncoding | Primary PCA | M30 | Whole mtDNA | McCrow et al. 2015 |
